# Signal-regulatory protein alpha is an anti-viral entry factor targeting viruses using endocytic pathways

**DOI:** 10.1371/journal.ppat.1009662

**Published:** 2021-06-07

**Authors:** Nicolás Sarute, Han Cheng, Zhonghao Yan, Karen Salas-Briceno, Justin Richner, Lijun Rong, Susan R. Ross

**Affiliations:** University of Illinois at Chicago College of Medicine, Chicago, Illinois, United States of America; Thomas Jefferson University, UNITED STATES

## Abstract

Signal-regulatory protein alpha (SIRPA) is a well-known inhibitor of phagocytosis when it complexes with CD47 expressed on target cells. Here we show that SIRPA decreased in vitro infection by a number of pathogenic viruses, including New World and Old World arenaviruses, Zika virus, vesicular stomatitis virus and pseudoviruses bearing the Machupo virus, Ebola virus and SARS-CoV-2 glycoproteins, but not HSV-1, MLV or mNoV. Moreover, mice with targeted mutation of the *Sirpa* gene that renders it non-functional were more susceptible to infection with the New World arenaviruses Junín virus vaccine strain Candid 1 and Tacaribe virus, but not MLV or mNoV. All SIRPA-inhibited viruses have in common the requirement for trafficking to a low pH endosomal compartment. This was clearly demonstrated with SARS-CoV-2 pseudovirus, which was only inhibited by SIRPA in cells in which it required trafficking to the endosome. Similar to its role in phagocytosis inhibition, SIRPA decreased virus internalization but not binding to cell surface receptors. We also found that increasing SIRPA levels via treatment with IL-4 led to even greater anti-viral activity. These data suggest that enhancing SIRPA’s activity could be a target for anti-viral therapies.

## Author summary

Viruses enter cells via different routes. Many RNA viruses require trafficking to a low pH compartment to accomplish entry. Similarly, phagocytosis of dead cells by macrophages results in their degradation in an acidic compartment. Here we show that SIRPA, which is a major inhibitor of phagocytosis, also inhibits infection by a variety of viruses that enter via acidic compartments, including many human pathogens such as Zika, Ebola and SARS-CoV-2. These findings suggest that phagocytosis and virus endocytosis share a common mechanistic pathway, and could lead to new approaches to the development of anti-viral therapeutics.

## Introduction

Virus entry is a major determinant of cellular tropism, host-range, and pathogenesis. Several host cell restriction factors limit virus entry when expressed on the cell membrane, like interferon-induced transmembrane proteins (IFITM), or when incorporated into virions, such as SERINC proteins [1–3]. A better understanding of how these factors influence the outcome of infection is important to our ability to develop prophylactic measures and therapeutics.

We previously showed that the E3 ubiquitin ligase Tripartite Motif 2 (TRIM2) in complex with Signal Regulatory Protein Alpha/Tyrosine-protein phosphatase non-receptor type substrate 1 (SIRPA/SHPS1), a negative regulator of phagocytosis, limited New World arenavirus (NWA) entry but did not alter infection by other viruses, such as the Old World arenaviruses (OWA) Lassa fever virus and lymphocytic choriomeningitis virus (LCMV), or the rhabdovirus vesicular stomatitis virus (VSV) [4]. Furthermore, we found that TRIM2 knockout reduced SIRPA-mediated inhibition of phagocytosis of apoptotic cells by mouse primary macrophages, thus mechanistically linking virus endocytosis and phagocytosis [4]. SIRPA is a receptor-type transmembrane glycoprotein with two cytoplasmic immunoreceptor tyrosine-based inhibition motifs (ITIM-1 and -2), which when phosphorylated recruit and activate the cytosolic SH2 domain-containing protein tyrosine phosphatases (SHP-1 and -2); these in turn dephosphorylate downstream substrates and activate additional kinases to shut off the engulfment program [5]. SIRPA’s phosphorylation status is relatively low in resting phagocytes but is greatly enhanced by binding to the membrane protein CD47 on targets such as tumor cells [6]. CD47-SIRPA interaction is thought to initiate a cascade that ultimately blocks F-actin-dependent phagocytosis [7]. Recently, it has also been shown that SIRPA inhibits integrin activation at the phagocytic synapse, thereby limiting the ability of macrophages to spread across the surface of the engulfment target [8]. CD47 is a “Don’t Eat Me” signal that protects healthy cells from macrophage engulfment but is highly expressed on tumor cells, thereby preventing their phagocytosis; it is thus a current target of anti-tumor therapies [9].

Here we report that unlike TRIM2, SIRPA has broad antiviral restriction activity in many different cell types and decreases infection by a variety of enveloped RNA viruses that require trafficking to an acidic compartment, including LCMV, VSV and the flavivirus Zika virus (ZIKV), as well as pseudotyped viruses bearing the glycoproteins of the coronavirus Severe Acute Respiratory Syndrome Coronavirus 2 (SARS-CoV-2), Machupo virus (MACV) and the filovirus Ebola virus (EBOV). Inhibition occurred at the internalization step of infection. SIRPA did not inhibit infection by two viruses that enter cells from the plasma membrane, herpes simplex virus-1 (HSV-1) and murine leukemia virus (MLV), or by mouse norovirus (mNoV), which enters cells via a cholesterol- and dynamin-dependent, but pH-, clathrin-, caveolae-, and macropinocytotic-independent pathway [10,11]. We also show that SIRPA’s cytoplasmic domain is needed for its antiviral activity in vitro and in vivo. Given the broad anti-viral activity of SIRPA, its function could be targeted to reduce virus infection.

## Results

### SIRPA is a broad antiviral host factor against enveloped RNA viruses

**Fig 1:**
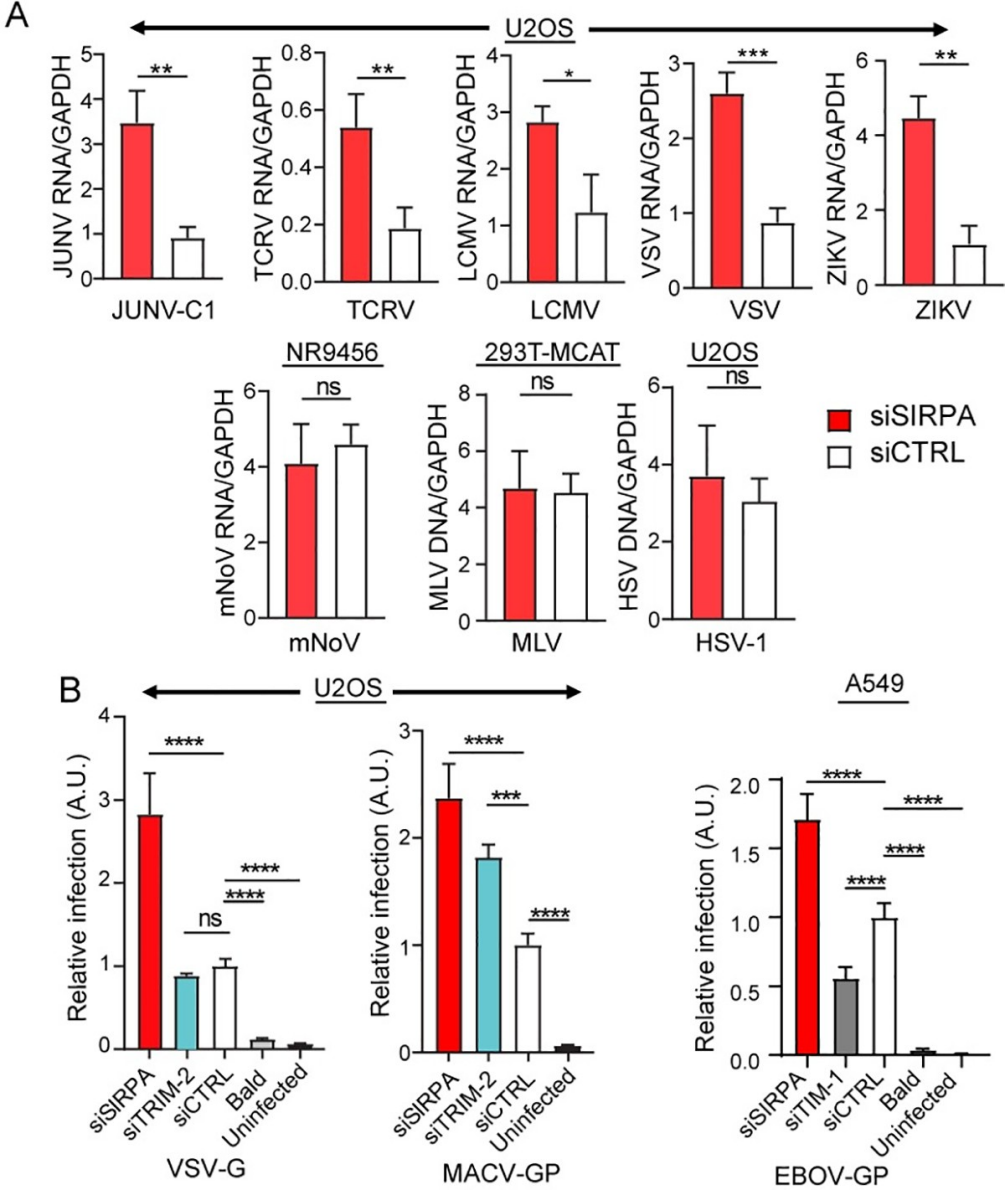
SIRPA has broad antiviral activity. A) U2OS, 293T and NR-9456 cells were transfected with the indicated siRNAs and infected with the indicated viruses 48 hours later. RNA was isolated at 14 (VSV, mNoV) or 24 (JUNV-C1, TCRV, LCMV, ZIKV) hpi, and DNA was isolated at 24 (HSV-1) or 48 (MLV) hpi. Viral nucleic acids were measured by qPCR. Shown is the average ± standard deviation (SD) of 3–6 independent experiments. Student’s T test was used to determine significance. **, *P* ≤ 0.005; ***, *P* ≤ 0.001; ns, not significant. B) U2OS and A549 cells were transfected with the indicated siRNAs and infected with pseudoviruses bearing the indicated viral glycoproteins. At 48 hpi, luciferase assays were performed. Bald viruses lacking any viral glycoprotein served as controls. Shown is the average ± SD of 3 independent experiments. One-way ANOVA was used to determine significance. **, *P* ≤ 0.001; ***, *P* ≤ 0.0003; ****, *P* ≤ 0.0001.

We previously identified SIRPA as a host restriction factor when testing which members of the TRIM2 interactome also diminished NWA infection [4]. We then asked whether SIRPA only limited entry by NWAs, like the vaccine strain of Junín virus Candid-1 (JUNV-C1) and Tacaribe virus (TCRV), or whether it also restricted viruses from other virus families not blocked by TRIM2. To assess SIRPA’s restriction activity, we used human or mouse small interfering RNAs (siRNA) that target the coding region to deplete SIRPA in the human cell lines U2OS and 293T and in the murine macrophage line NR-9456. These cell lines were chosen for their known ability to be infected with the different viruses, and because they expressed SIRPA (S1A Fig). We infected the siRNA-transfected U2OS cells with the NWAs JUNV-C1 and TCRV, the OWA LCMV, the rhabdovirus VSV, the flavivirus ZIKV and the herpesvirus HSV-1. Additionally, 293T cells stably expressing mouse cationic amino acid transporter 1 (mCAT-1), the receptor for the retrovirus murine leukemia virus (MLV) [12], were infected with MLV, and NR-9456 cells with murine norovirus (mNoV). Infection levels were determined by quantifying viral RNA or DNA at different timepoints after infection by real-time RT-qPCR. We found that depletion of SIRPA increased infection by JUNV-C1, TCRV, LCMV, VSV and ZIKV, but did not affect infection by mNoV, MLV or HSV-1 (Fig 1A).

We also determined whether SIRPA restricted entry of pseudoviruses bearing the glycoproteins of several human pathogenic viruses. We pseudotyped lentivirus- or MLV-based luciferase-containing viral vectors with the filovirus EBOV and the NWA Machupo virus (MACV) glycoproteins (GP), as well as pseudoviruses bearing the VSV glycoprotein (G); bald pseudoviruses lacking any viral glycoprotein served as controls. We infected the lung cancer epithelial cell line A549 with EBOV GP and U2OS cells with VSV G and MACV GP pseudoviruses, after siRNA-mediated knockdown of SIRPA (S1B Fig). Again, the cell lines were chosen for their known ability to be infected with the different pseudoviruses and for SIRPA expression (S1B Fig). Knockdown of TIM-1 and TRIM2 served as controls for these experiments; TIM-1 enhances EBOV entry [13,14] while TRIM2 restricts MACV entry [15]. We found that SIRPA limited infection of EBOV GP, MACV GP and VSV G pseudoviruses while knockdown of TRIM2 only affected infection by MACV and of TIM-1 by EBOV pseudotypes (Fig 1B).

These data suggest that SIRPA reduces infection by viruses that require trafficking to acidic endosomes (NWAs, OWAs, VSV, ZIKV, EBOV), but not viruses that fuse at the plasma membrane (MLV, HSV-1) or whose entry is pH-independent (mNoV). Moreover, SIRPA-mediated inhibition occurred in U2OS (osteosarcoma), 293T (kidney epithelia) and A549 (lung epithelia), suggesting that this inhibition is not restricted to a specific cell type.

### SIRPA restricts endocytosis-mediated but not plasma membrane entry of SARS-CoV-2

**Fig 2:**
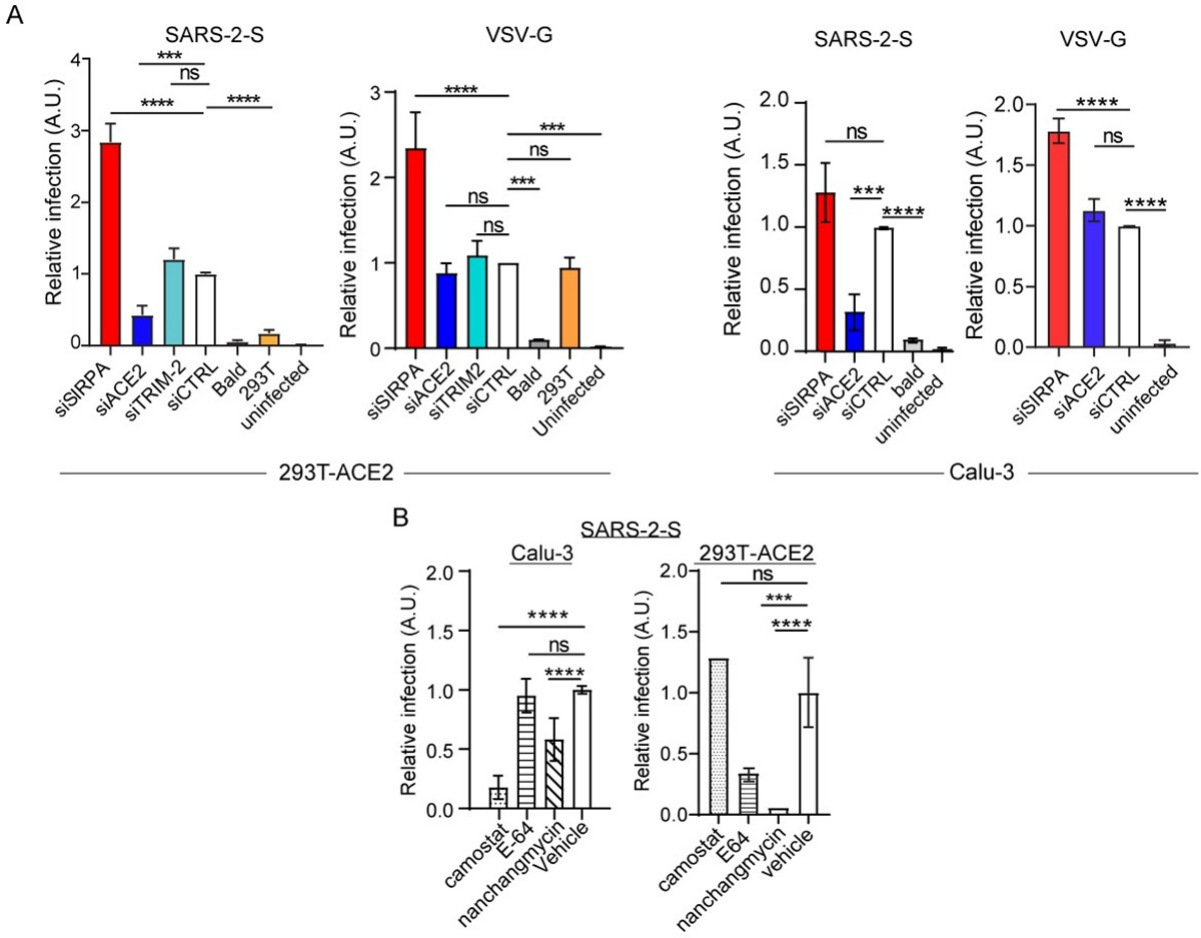
SIRPA inhibits endosomal but not cell surface entry of SARS-CoV-2. A) 293T-ACE2 or Calu-3 cells were transfected with the indicated siRNAs and then infected with SARS-2-S or VSV G pseudoviruses. Luciferase assays were performed at 48 hpi. B) Calu-3 or 293T-ACE2 cells were treated with the indicated drugs (camostat inhibits TMPRSS2; E-64D inhibits endosomal Cat B/L; nanchangmycin is an endocytosis inhibitor) infected with SARS-2-S and VSV G pseudoviruses. Shown is the average ± SD of 3–6 independent experiments. One-way ANOVA was used to determine significance. *, *P* ≤ 0.02; **, *P* ≤ 0.001; ***, *P* ≤ 0.0003; ****, *P* ≤ 0.0001.

All the viruses whose entry was inhibited by SIRPA share in common entry from an acidic endocytic pathway. The novel coronavirus SARS-CoV-2 uses this endocytic pathway in some cell types but not others. In 293T cells stably expressing its cellular receptor angiotensin I converting enzyme 2 (ACE2) (293T-ACE2), SARS-CoV-2 binding to the receptor is followed by endocytosis and trafficking to the late endosome, where cleavage by cathepsins B or L (Cat B/L) primes the spike (S) protein for fusion [16,17]. In other cell types, such as lung epithelia, SARS-CoV-2 fusion occurs at the plasma membrane after binding to ACE2, because the S protein is cleaved by the membrane serine protease Transmembrane Protease, Serine 2 (TMPRSS2) [16]. To determine if SIRPA inhibition of SARS-CoV-2 infection was dependent on the entry pathway, we first showed that SIRPA was expressed in 293T-ACE2 cells and in the lung epithelial cell line Calu-3, which expresses TMPRSS2 (S2A Fig). We also demonstrated SIRPA knockdown in both cell lines (S2B Fig). We then infected Calu-3 and 293T-ACE2 cells with SARS-2-S and control VSV-G pseudoviruses after knockdown of SIRPA and ACE2 and measured infection by luciferase assays. SIRPA knockdown increased infection by SARS-2-S pseudoviruses in 293T-ACE2 cells (Fig 2A). However, SIRPA knockdown did not significantly increase SARS-2-S pseudovirus infection in Calu-3 cells, while ACE2 knockdown greatly decreased infection in both cell types (Fig 2A). VSV G pseudovirus infection was enhanced by SIRPA but was not affected by ACE2 knockdown in Calu-3 and 293T-ACE2 cells (Fig 2A). Only VSV G but not SARS-2S pseudoviruses infected 293T cells lacking ACE2 and neither pseudovirus was affected by TRIM2 knockdown. Bald viruses lacking viral glycoproteins did not infect either cell type.

As controls for these experiments, we used small molecule inhibitors of the two SARS-CoV-2 infection pathways: camostat mesylate, a serine protease inhibitor that targets TMPRSS2; E-64D, an inhibitor of endosomal Cat B/L; and nanchangmycin, a broad inhibitor of endocytosis. As shown by others, we confirmed that camostat greatly reduced SARS-2-S infection levels in Calu-3 cells but not in 293T-ACE2 cells. Conversely, E-64D decreased infection in 293T-ACE2 but not in Calu-3 cells (Fig 2B) [16, 17]. Nanchangmycin had a modest effect on Calu-3 infection and almost completely abrogated SARS-2-S infection in 293T-ACE2 cells (Fig 2B). SARS-2-S infection of Calu-3 cells was inhibited but not completely abrogated by camostat, which likely reflects residual S protein priming by endosomal Cat B/L, as was previously described (Fig 2B) [16]. Thus, the existence of two independent entry pathways for SARS-CoV-2 allowed us to confirm that SIRPA targets the pathway and not the particular virus.

### SIRPA’s cytoplasmic domain is needed for antiviral activity

**Fig 3:**
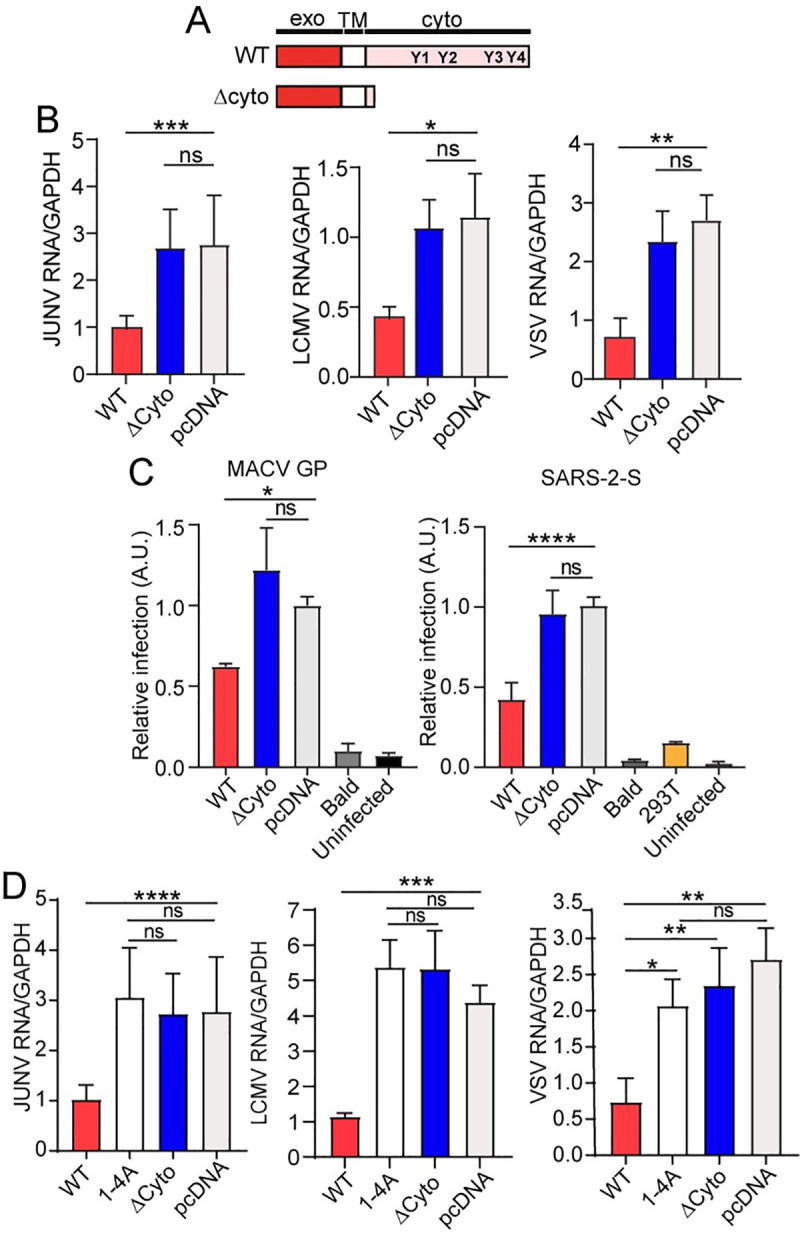
SIRPA’s cytoplasmic tail is required for its antiviral activity. A) Top: Diagram of WT and truncated (ΔCyto) human SIRPA constructs. Y1 through Y4 represent the tyrosines needed for SIRPA’s anti-phagocytic activity. B) U2OS cells were transfected with the indicated plasmids and 24 hr later, infected with the indicated viruses. At 24 hpi, RNA was isolated and examined by RT-qPCR for infection levels. Shown is the average ± SD of 3–10 independent experiments. One-way ANOVA was used to determine significance. *, *P* ≤ 0.015; **, *P* ≤ 0.003; ***, *P* ≤ 0.0002. C) 293T-ACE2 cells were transfected with the indicated plasmids and 24 hours later, infected with the indicated pseudoviruses. At 48 hpi, luciferase assays were performed. Shown is the average ± SD of 3–4 independent experiments. One-way ANOVA was used to determine significance. *, *P* ≤ 0.01; ****, *P* ≤ 0.0001. D) The four Y residues in SIRPAs cytoplasmic tail were mutated to A residues. Experiments were carried out as in panel B. Shown is the average ± SD of 3–4 independent experiments. One-way ANOVA was used to determine significance. **, *P* ≤ 0.002; ****, *P* ≤ 0.0001.

CD47/SIRPA binding results in SIRPA’s interaction with other proteins and downstream signaling that is governed by its cytoplasmic domain [5]. We next tested whether the cytoplasmic domain was also required for SIRPA inhibition of virus infection. We overexpressed a FLAG-tagged expression construct encoding human SIRPA (WT) and a mutant construct lacking most of the cytoplasmic domain (ΔCyto) (Fig 3A) and tested their effect on infection with replication-competent viruses and pseudoviruses in U2OS and 293T-ACE2 cells, respectively. Both the WT and ΔCyto constructs were expressed at similar levels in U2OS and 293T-ACE2 cells (S3A Fig). At 24 hours (hr) post-transfection, we infected the transfected U2OS cells with JUNV-C1, LCMV and VSV and assessed viral RNA levels. We also tested the constructs in 293T-ACE2 cells and at 24 hr. post-transfection, infected the cells with MACV GP and SARS-2-S luc-pseudoviruses; bald viruses lacking viral glycoproteins served as controls, as did 293T cell lacking ACE2. Over-expression of SIRPA WT reduced infection by JUNV-C1, LCMV and VSV viruses (Fig 3B) and pseudoviruses bearing viral glycoproteins from MACV and SARS-CoV-2 (Fig 3C), while infection levels in ΔCyto-expressing cells were similar to those observed for the pcDNA empty vector control (Fig 3B and 3C).

To further investigate the role of SIRPA’s cytoplasmic domain in infection, we made a SIRPA construct containing alanine (A) replacements for all four tyrosine residues in the ITIMs. The 1-4A mutant was expressed at similar levels to the WT and ΔCyto constructs after transfection into U2OS cells (S3B Fig). We tested the ability of the 1-4A mutant to block infection of JUNV-C1, LCMV and VSV after transfection into U2OS cells; as was observed for ΔCyto, the 1-4A mutant failed to suppress infection by any of the viruses (Fig 3D).

### SIRPA restricts in vivo virus infection

**Fig 4:**
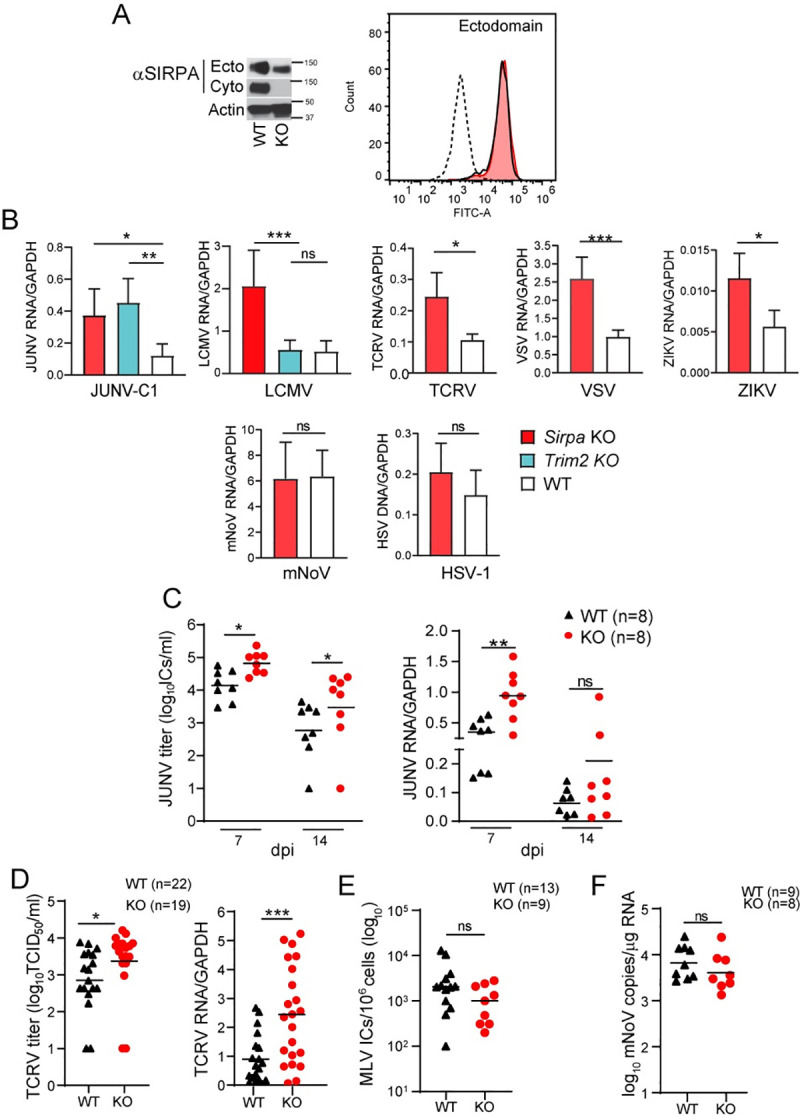
SIRPA KO renders cells and mice more susceptible to virus infection. A) SIRPA expression in BMDMs isolated from WT and SIRPA KO mice was analyzed in total cell extracts by western blot, using antibodies to the ectodomain or cytoplasmic tail and by FACS, using antibody to the ectodomain. The molecular weight (MW) of mouse SIRPA is 110kD. B) BMDMs isolated from mice of the indicated genotype were infected with the specified viruses and RNA was isolated at 14 hpi (VSV, mNoV), 24 hpi (JUNV-C1, TCRV, LCMV) or 48 hpi (ZIKV). HSV-1 DNA was isolated at 24 hpi. Viral nucleic acids were examined by qPCR for infection levels. Shown is the average ± SD of 4–6 independent experiments. One-way ANOVA was used to determine significance. *, *P* ≤ 0.02; **, *P* ≤ 0.002; ***, *P* ≤ 0.0005. C) Adult mice of the indicated genotype were infected with JUNV-C1 by IP injection, and at 7 or 14 dpi, virus and RNA was isolated from spleens and analyzed for titers and by qPCR. Each point represents an individual mouse. Shown is the average ± SD. Student’s T test with Welch’s correction was used to determine significance. *, *P* ≤ 0.01; **, *P* ≤ 0.004. D) Newborn WT and SIRPA KO mice were infected with TCRV by IP injection. Viral titers and RNA levels in spleen were analyzed at 7 dpi. Student’s T test with Welch’s correction was used to determine significance. *, *P* ≤ 0.01; ***, *P* ≤ 0.001. E) Newborn mice were infected with 2000 ICs of MLV and virus titers in spleen were measured at 16 dpi. F) WT and SIRPA KO mice were orally infected with mNoV strain CW3 and 3 dpi viral RNA levels in spleens were examined. Unpaired t test with Welch’s correction was used to determine significance.

To determine if SIRPA also had antiviral activity in vivo, we used a *Sirpa* mutant mouse (B6.129P2 Sirpa^<tm1Nog>/Rbrc^) that has a targeted deletion of the cytoplasmic domain identical to the ΔCyto human SIRPA construct and is a functional knockout (referred to as KO for the remainder of the manuscript) (Fig 4A) [18]. SIRPA KO mice are viable and morphologically normal, although they are reported to have lower levels of platelets and T cells and their macrophages have increased phagocytic activity [19]. We first analyzed SIRPA expression in bone marrow-derived macrophages (BMDM) from 8-12-week-old SIRPA KO mice and WT littermates by western blot (total) or flow cytometry (surface), using antibodies that recognize the ectodomain and cytoplasmic tail. As previously reported, the levels of total protein were decreased in SIRPA KO BMDMs with respect to WT cells, although surface levels were similar (Fig 4A) [20]. No protein was detected in SIRPA KO cells when an antibody that recognizes the cytoplasmic domain was used in western blots (Fig 4A).

We then analyzed SIRPA restriction activity ex vivo by infecting BMDMs from SIRPA KO and WT mice with JUNV-C1, TCRV, LCMV, VSV, ZIKV, mNoV, and HSV-1, all of which can infect mouse cells. As controls, we infected BMDMs from TRIM2 KO mice with JUNV-C1 and LCMV, since we previously showed that BMDMs from these mice were more highly infected by NWAs but not OWAs [4]. Similar to what was seen with SIRPA knock-down cells, SIRPA KO primary cells were more highly infected than WT cells by JUNV-C1, TCRV, LCMV, VSV, and ZIKV, but not mNoV or HSV-1 (Fig 4B). Only JUNV-C1 and not LCMV showed higher infection of TRIM2 KO cells (Fig 4B).

To determine whether SIRPA limits virus replication in vivo, we infected SIRPA KO and WT mice with the NWAs JUNV-C1 and TCRV, and with MLV and mNoV as controls. Eight-to-twelve-week-old mice were inoculated intraperitoneally with JUNV-C1, and viral RNA and titers in the spleen were assessed at 7- and 14-days post infection (dpi). At 7 dpi, JUNV-C1 titers were significantly higher for the SIRPA KO mice with respect to the WT littermates, as were RNA levels (Fig 4C). Mice clear JUNV-C1 infection around 14 dpi [21]; thus, we also analyzed that time point to determine if the higher level of virus infection in the SIRPA KO mice persisted at later times. The SIRPA KO mice were also significantly more infected than were WT mice at 14 dpi, although virus titers decreased on average 10-fold for both genotypes (Fig 4C). TCRV in vivo infections were carried out by infecting newborn mice (1–3 days old) by intraperitoneal injection. TCRV infection levels in spleen were assayed at 7 dpi and as was seen for JUNV-C1 infections, the SIRPA KO pups showed significantly higher levels of viral RNA and titers in comparison to the WT mice (Fig 4D).

Newborn SIRPA KO and WT mice were also infected with MLV, and infection was analyzed at 16 dpi by measuring virus titers in spleen, a technique commonly used in the field [22,23]. Both mouse strains showed similar levels of infection (Fig 4E). Lastly, SIRPA KO and WT mice (6–8 weeks old) were orally infected with mNoV and spleen RNA was analyzed at 3 dpi. As expected, there were no differences in viral RNA levels between mice of both genotypes (Fig 4F). Thus, SIRPA controls both in vitro and in vivo infection by viruses using the acidic endosomal pathway for entry.

### Integrin activation but not SIRPA-CD47 interaction bypasses SIRPA antiviral activity

**Fig 5:**
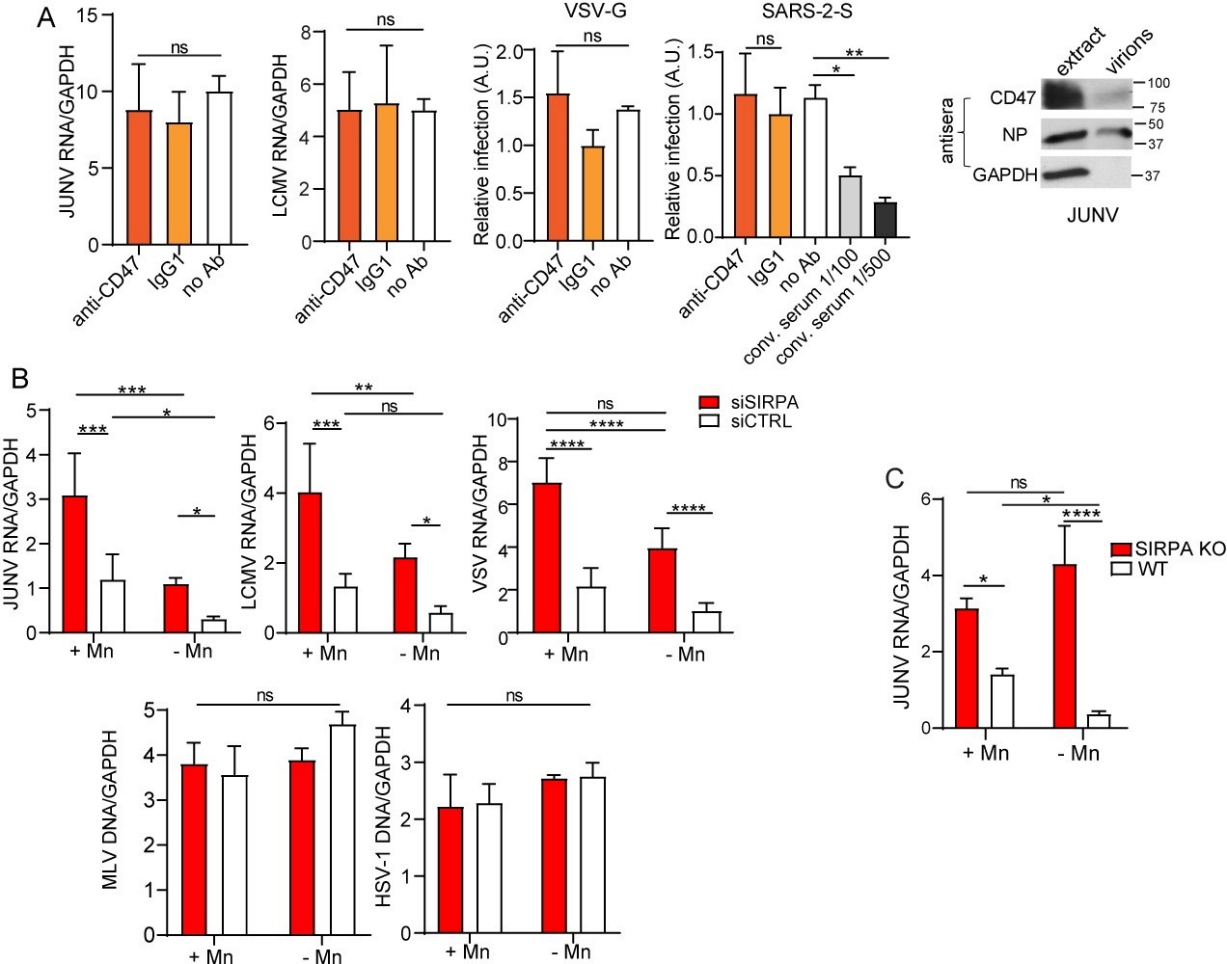
Integrin activation, rather than interaction with CD47, modulates SIRPA antiviral activity. A) Viruses (JUNV-C1, LCMV) and pseudoviruses (VSV-G, SARS-2-S) were incubated with the indicated antibodies for 1 hr on ice before being added to U2OS and 293T-ACE2 cells, respectively. RT-qPCR for viral RNA or luciferase assays were carried out at 24 and 48 hpi, respectively. The data shown represent the average ± SD of 2 (convalescent serum) - 4 independent experiments. One-way ANOVA was used to determine significance. *, *P* ≤ 0.02; **, *P* ≤ 0.002. Panel on the right shows western blot analysis of JUNV-C1-infected U2OS cell extracts and virions (5E5 ICs) probed with the same anti-CD47 antibody, anti-Junín virus NP monoclonal antibody or anti-GAPDH. B) U2OS and 293T-mCAT cells were transfected with the indicated siRNAs and 48 hours later were treated with 1mM MnCl_2_ for 2 hr. After MnCl_2_ treatment, U2OS cells were infected with JUNV-C1, LCMV, VSV, MLV and HSV-1 for 24 hr and 293T-mCAT cells with MLV for 48 hr. Viral RNA and DNA levels were determined by qPCR. The data shown represent the average ± SD of 3–4 independent experiments. Two-way ANOVA was used to determine significance. *, *P* ≤ 0.05; **, *P* ≤ 0.005: ***, *P* ≤ 0.0004; ***, *P* ≤ 0.0001. C) Primary fibroblasts isolated from WT and SIRPA KO mice were treated with 1mM MnCl_2_ for 2 hr and infected with JUNV-C1 for 24 hr. Viral RNA was examined by RT-qPCR. Shown is the average ± SD of 3 independent experiments. Two-way ANOVA used to determine significance. *, *P* ≤ 0.02; ****, *P* ≤ 0.0001.

SIRPA-CD47 interaction inhibits different engulfment signals at the phagocytic synapse [24]. We next tested whether CD47 on the virion surface, which could be acquired during budding from host cells, limited viral endocytosis by SIRPA-expressing cells. The anti-CD47 monoclonal antibody B6H12, which blocks phagocytosis, was used to determine the level of CD47 surface expression on 293T and U2OS cells by flow cytometry; both expressed CD47 (S4 Fig). We used 293T cells to prepare SARS-2-S and VSV-G pseudoviruses while JUNV-C1 and LCMV were isolated from infected U2OS cells. JUNV-C1 and LCMV are typically isolated from Vero E6 (monkey) and BHK-21 (hamster) cells, respectively, for which blocking anti-CD47 antibodies were not available. Western blot analysis of the JUNV-C1 virus preparation using the B6H12 antibody demonstrated incorporation of CD47 in virions (Fig 5A). We then incubated both the pseudoviruses and viruses with B6H12 and tested whether infection was blocked. There was no difference in infection levels between the viruses treated with the anti-CD47 antibody compared to those that were untreated or incubated with an isotype control (Fig 5A). In contrast, convalescent serum from rhesus macaques infected with SARS-CoV-2, strongly inhibited infection of SARS-2-S pseudoviruses (Fig 5A).

It was recently shown that SIRPA inhibits inside-out activation of integrins at the phagocytic synapse to limit macrophage spreading across the surface of the engulfment target and ingestion by phagocytosis, and that activation of integrins by MnCl_2_ treatment, which locks α integrins in an open conformation, bypassed SIRPA-mediated inhibition and rescued engulfment [8]. We next determined whether integrin activation by MnCl_2_ would also bypass SIRPA’s antiviral activity. U2OS cells were transfected with siSIRPA or siCTRL, treated with MnCl_2_ and infected with JUNV-C1, LCMV, VSV, MLV or HSV-1. MnCl_2_ treatment enhanced JUNV-C1, LCMV and VSV but not MLV or HSV-1 infection in both siSIRPA- and siCTRL- S5A and S5B Fig). As a result, JUNV-C1, LCMV and VSV, but not HSV-1 or MLV infection was greatly increased by co-treatment with SIRPA siRNA and MnCl_2_ (Fig 5B).

We also examined JUNV-C1 infection in primary fibroblasts from WT and SIRPA KO mice treated with MnCl_2_. MnCl_2_ treatment increased infection in WT cells, but did not further enhance infection in SIRPA KO fibroblasts (Fig 5C). MnCl_2_ treatment also did not increase phagocytosis of apoptotic cells by SIRPA knockout cells (S6A and S6B Fig). This may be because unlike knockdown cells, which still have residual SIRPA activity, knockout cells express no functional SIRPA. Thus, maximal levels of infection and phagocytosis may be already achieved that cannot be further enhanced by MnCl_2_. These results are consistent with a SIRPA-mediated inhibition of integrin activation, leading to decreased phagocytosis and virus infection.

### SIRPA limits viral internalization but not binding to the cell surface

We previously showed that TRIM2 expression did not affect virus binding to the cells and that it limited endocytosis of NWAs in human and mouse cells [4]. Unlike TRIM2, which is cytoplasmic, SIRPA is a transmembrane protein with a large extracellular domain and its expression could affect virus binding to cells. To test this, infectious TCRV virions and SARS-2-S pseudoviruses were labeled with FITC and virus binding to U2OS and 293T-ACE2 cells, respectively, was quantified by flow cytometry after siRNA knockdown of SIRPA. We used TCRV in this assay, because we previously showed that depletion of the voltage gated calcium channel (VGCC) receptor subunits α1s, α2δ2, and β3 reduced virus binding; the VGCC is the cellular receptor for TCRV on human cells [25]. SIRPA knockdown did not alter TCRV binding to U2OS cells, while knockdown of the VGCC had a profound effect on binding (S7A Fig). SARS-2-S pseudovirus binding was also assessed upon siRNA knockdown of SIRPA and ACE2. As was seen for infectious TCRV, SIRPA expression levels did not alter SARS-2-S pseudovirus binding to cells, while ACE-2 knockdown significantly decreased binding, to similar levels as 293T cells not expressing ACE-2 (S7A Fig).

**Fig 6:**
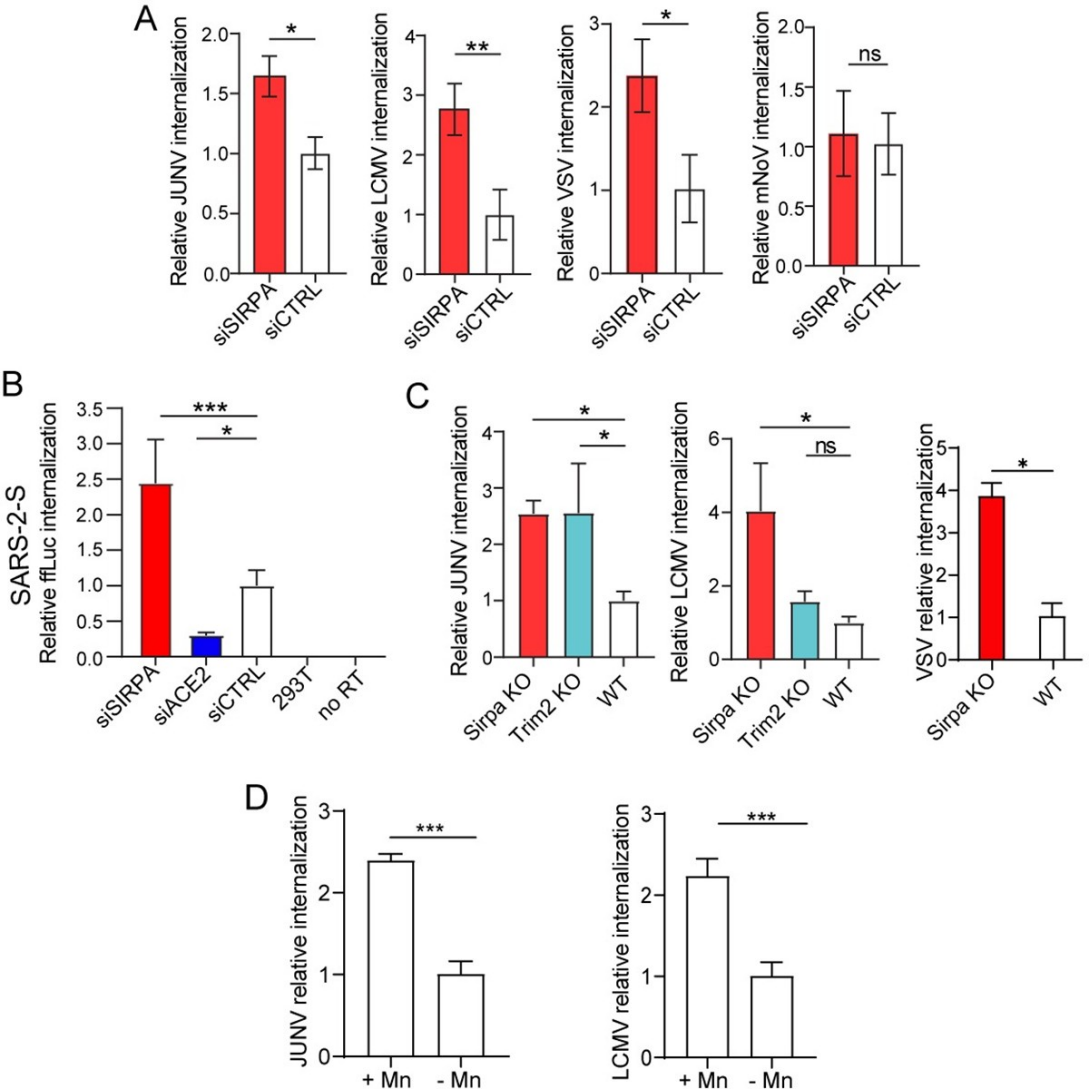
SIRPA inhibits virus internalization. A) Viral internalization assays (VIA) were carried out with U2OS or NR-9456 cells transfected with the indicate siRNAs. Internalized viral RNA was analyzed by RT-qPCR. Shown is the average ± SD of 3 independent experiments. Student’s T test was used to determine significance. *, *P* ≤ 0.02; **, *P* ≤ 0.007; B) VIAs were performed with SARS-2-S pseudoviruses and 293T-ACE2 cells transfected with the indicated siRNAs. RT-qPCR for the luciferase gene was used to measure internalized pseudovirus. 293T cells and no reverse transcriptase (no RT) served as controls. Shown is the average ± SD of 3 independent experiments. One-way ANOVA was used to determine significance. *, *P* ≤ 0.02; ***, *P* ≤ 0.001. C) VIAs were performed with BMDMs isolated from mice of the indicated genotypes with JUNV-C1, LCMV or VSV. Shown is the average ± SD of 3 independent experiments. One-way ANOVA was used to determined significance. *, *P* ≤ 0.03; **, *P* ≤ 0.007. D) MnCl_2_ treatment increases virus internalization in ear fibroblasts. Shown is the average ± SD of 3 experiments. Student T test was used to determine significance. ***, *P* ≤ 0.0002.

We next performed virus internalization assays (VIA) using the infectious viruses JUNV-C1, LCMV, VSV and mNoV to determine whether SIRPA affected this step. After binding on ice for 1 hr, internalization was allowed to proceed for 1 hr at 37°C, virus was stripped from cells and internalized viral RNA was quantified by RT-qPCR. We found that SIRPA knockdown resulted in increased internalization of JUNV-C1, LCMV, and VSV, but did not affect internalization of mNoV (Figs 6A and S7B). We also tested SARS-2-S pseudovirus internalization in SIRPA-depleted 293T-ACE2 cells using primers to the firefly luciferase (ffLuc) gene found in the reporter virus genome. The levels of internalized ffLuc were increased in SIRPA-depleted cells and, as expected, reduced upon ACE2 knockdown (Figs 6B and S7B).

We also performed JUNV-C1, LCMV and VSV VIAs with SIRPA KO and WT BMDMs; cells from TRIM2 KO mice served as a control for the arenavirus infections. As was observed with the in vitro knockdowns, the level of internalized viral RNA was significantly higher in SIRPA KO cells for JUNV-C1, LCMV and VSV, and in TRIM2 KO cells for JUNV-C1, confirming the role of SIRPA in limiting viral internalization (Fig 6C).

Finally, to examine whether integrin activation would increase virus internalization, we treated primary ear fibroblasts from WT mice with MnCl_2_ and performed VIAs with JUNV-C1 and LCMV. MnCl_2_ treatment increased internalization of both viruses (Fig 6D).

These data suggest that SIRPA is restricting virus entry by acting at a post-binding step to limit virus internalization and that this restriction could be decreased by integrin activation.

### Interleukin-4 treatment increases SIRPA expression, thereby decreasing virus internalization

**Fig 7:**
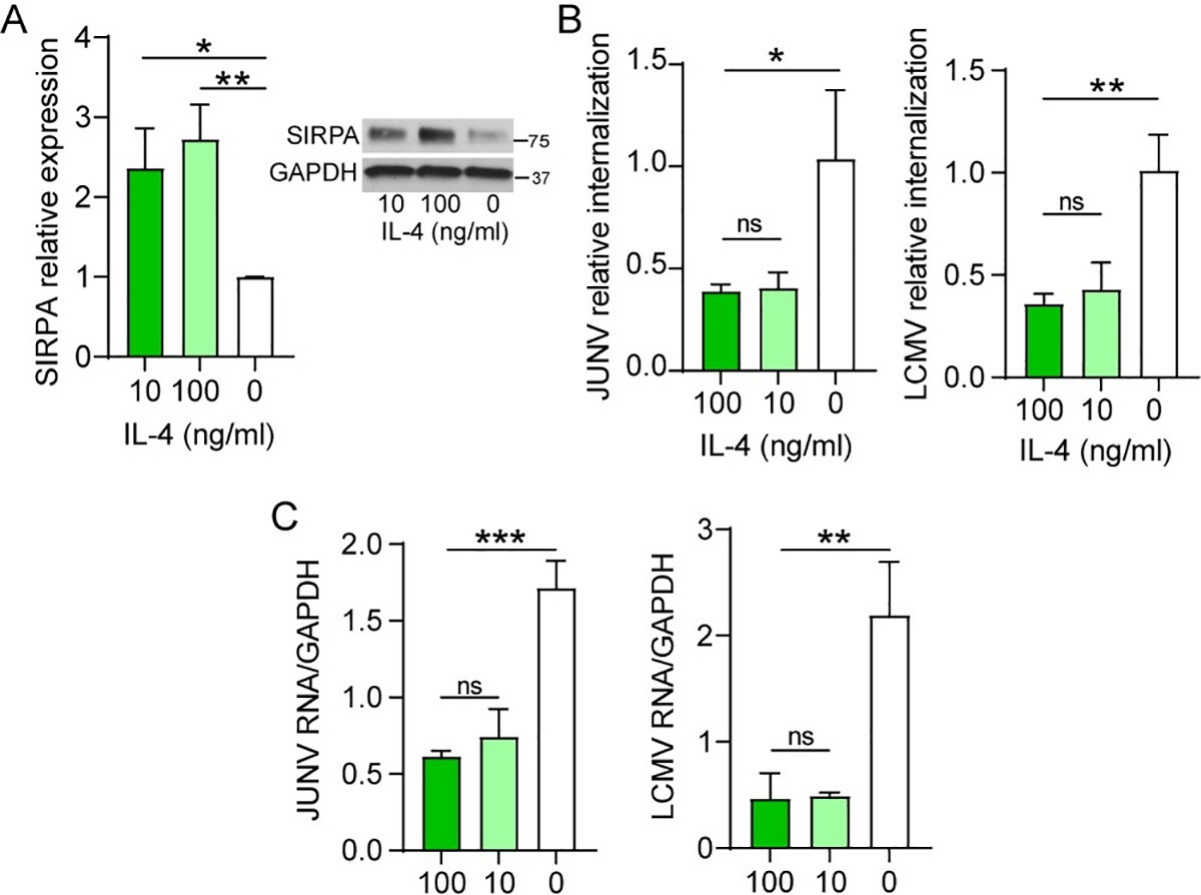
Interluekin-4 (IL-4) treatment upregulates SIRPA expression and decreases viral internalization. A) U2OS cells were pre-treated with the indicated concentrations of IL-4 for 48 hr and SIRPA expression levels were quantified by western blot. The MW of human SIRPA is 85kD. Shown is the average ± SD of 3 independent experiments. One-way ANOVA was used to determine significance. **, P ≤ 0.003. B) and C) U2OS cells were pre-treated with IL-4 and were subjected to VIAs (B) and infection for 24 hr. (C) with JUNV-C1 and LCMV. Shown is the average ± SD of 3–4 independent experiments. One-way ANOVA was used to determine significance. *, *P* ≤ 0.02; **, *P* ≤ 0.002; ***, *P* ≤ 0.0005.

SIRPA expression levels increase upon interleukin-4 (IL-4) treatment in vitro [26]. Thus, we determined whether IL-4 treatment would enhance SIRPA’s anti-viral activity. We treated U2OS cells with different concentrations of IL-4 and performed VIAs and infections with JUNV-C1 and LCMV. IL-4 treatment increased total and surface levels of SIRPA protein by ~2-fold in comparison to untreated cells (Figs 7A, S8A and S8B). This resulted in reduced internalization of both JUNV-C1 and LCMV (Fig 7B). Analysis of viral infection at 24-hours post-infection (hpi) showed an even higher reduction in viral RNA levels upon IL-4 treatment (Fig 7C), which may be due to anti-viral activities of IL-4 that occur at later steps in infection.

## Discussion

The diversity of entry mechanisms and pathways exploited by viruses to invade and replicate in host cells has led to the identification of many cellular proteins that act as restriction factors at different steps in infection. The best-known host factors that work by inhibiting virus entry are members of the IFITM family, which were discovered in a functional genomic screen for factors that affected influenza A virus infection [27]. Subsequent studies showed that IFITM1, 2 and 3, which are transmembrane proteins, function against diverse virus families by blocking virus-cell fusion [28,29]. Unlike IFITM proteins, which blocks the fusion of viruses after they traffic to the endosome, SIRPA works by blocking endocytosis of those viruses that require trafficking to this compartment. These data suggest that the initial step of invagination/endocytosis for viruses that traffic to acidic compartments is mechanistically similar and independent of both the entry receptor and pathway (clathrin-mediated, caveola, macropinocytosis, etc.). SIRPA interaction with CD47 on target cells decreases phagocytosis by about 50%, which is similar to its effect on virus infection. IFITM proteins also diminish but do not completely block infection. Thus, SIRPA, like IFITM proteins, is likely a modulator of viral infection.

Many viruses traffic to acidic endosomes for entry. NWAs, rhabdoviruses and flaviviruses use clathrin-mediated endocytosis as their predominant route of entry. Receptor-mediated entry of OWAs involves macropinocytosis, where viruses pass through an unknown early endosomal compartment, followed by their delivery to acidified late endosomes. Filoviruses are also internalized by macropinocytosis upon binding to cells and then traffic to the endo-lysosomal compartment, where they require cathepsin cleavage and low pH for fusion and entry [30–34]. Some, like arenaviruses, require low pH to trigger virus-cell fusion, while others, like EBOV, require cleavage of their glycoprotein by endosome-resident proteases such as cathepsins as well as low pH [35]. Although noroviruses exploit endocytic routes to gain entry into cells, mNoV entry is cholesterol- and dynamin-dependent but pH-independent [36]. On the other hand, herpesviruses and retroviruses like MLV fuse at the cell surface to deliver viral capsids into cells [37,38].

Interestingly, SARS-CoV-2, whose S protein requires proteolytic cleavage to initiate fusion, enters from the plasma membrane in cells that express TMPRSS2 on the surface, while in cells lacking this cell-surface protease, it traffics to the cathepsin-containing late endosome. The use of SARS-2-S pseudotypes in our experiments thus gave us an excellent system with which to test the role of entry pathway on SIRPA’s ability to inhibit infection. Indeed, SIRPA only restricted infection of 293T-ACE2 cells, in which trafficking to the cathepsin-containing acidic endosomes is required for entry, but not of Calu-3 lung epithelial cells, which express both ACE2 and TMPRSS2 on the surface (Fig 2A) [16]. Lung epithelia are a major target of SARS-CoV-2 infection in vivo, and it was recently shown that other members of the TMPRSS family (mostly TMPRSS13 and at a lesser extent 11D, 11E and 11F) are able to prime the S protein for fusion in these cells. However, TMPRSS proteases are not expressed at detectable levels in different extrapulmonary targets of SARS-CoV-2, such as cardiomyocytes and macrophages, which express both ACE2 and SIRPA [39–41]. Whether SIRPA also restricts infection in these cell types and limits SARS-CoV-2 infection in vivo remains to be tested.

SIRPA is a transmembrane glycoprotein with two ITIMs in the cytoplasmic domain, which upon phosphorylation recruit and activate SHP tyrosine phosphatases to trigger downstream signaling and ultimately phagocytosis, although the exact targets are not well-defined [5]. To determine whether SIRPA’s cytoplasmic domain was needed for its anti-viral activity, we tested constructs lacking the cytoplasmic domain (ΔCyto) and with the four tyrosine in the ITIMs mutated to alanine (1-4A). Both constructs failed to inhibit infection by JUNV-C1, LCMV and VSV (Fig 3D). Ex vivo infection of primary cells derived from SIRPA KO mice, which similarly lack most of the cytoplasmic domain, confirmed the phenotype observed in vitro (Fig 4A and 4B). Interestingly, SIRPA KO mice showed higher JUNV-C1 infection levels at later timepoints as well, suggesting that SIRPA contributes to diminution of viral spread in vivo (Fig 4C). Future studies will determine whether SIRPA’s inhibition of virus endocytosis is due to structural requirements of the cytoplasmic domain or signal transduction.

Enveloped viruses commonly acquire host proteins in their membranes during budding, which they use to exploit cellular pathways, enhance their infectivity or evade the antiviral immune response [42,43]. Because binding of SIRPA on phagocytes to CD47 on target cells inhibits phagocytosis, we tested whether CD47 on the virion surface, potentially acquired during budding, could bind to SIRPA and limit viral endocytosis. Incubation of viruses and pseudoviruses produced in CD47-expressing cell lines with a blocking anti-CD47 monoclonal antibody did not affect infection levels (Figs 5A and S4). Thus, unlike its role in phagocytosis, SIRPA-mediated restriction of virus entry probably involves a CD47-independent mechanism.

SIRPA also modulates the activity of integrins at the phagocytic synapse by inhibiting their inside-out activation, which is required for phagocytosis [8]. Viruses from different families exploit activated integrins to infect cells by physically interacting through RGD (Arg-Gly-Asp) or other non-RGD binding motifs displayed on their envelope glycoproteins, or by the incorporation of carbohydrates [44]. For example, HSV-2 exploits αvβ3 integrins to enhance its binding to susceptible cells [45]. In contrast, we showed that integrin activation does not affect virus binding, but instead enhances the process of virus internalization and that this is limited to viruses that traffic to acidic compartments for entry. At later timepoints, MnCl_2_ may also enhance infection by promoting virus replication; for example, viral RNA polymerases require divalent metal ions and both the OWA Lassa virus RNA polymerase and MLV reverse transcriptase have higher synthetic rates in the presence of Mn than Mg ions [46,47]. We suggest that SIRPA restricts virus internalization an early step in infection by limiting integrin activation, which similar to phagocytosis may be required to allow virus engulfment. How this occurs mechanistically is currently being investigated.

For the viruses tested here, SIRPA may be a feasible target to limit entry. Many of the viruses inhibited by SIRPA, such as filo- and arenaviruses, are thought to first infect sentinel cells of the immune system such as macrophages and from there to spread to other tissues, ultimately initiating cytokine cascades that result in pathogenesis [48]. Slowing infection at an early stage has the potential to keep virus levels low while the immune system response is activated. Viruses such as the NWAs, OWAs, Zika and Ebola virus, induce cytokines early in infection, through interaction with pathogen recognition receptors (PRRs) [21,49–52]. SIRPA expression is induced by cytokines such as IL-4. Thus, SIRPA may be part of the arsenal of host-anti-virus factors induced by PRR activation. In a proof-of-concept experiment, we showed that IL-4 treatment enhanced SIRPA expression in vitro, thereby restricting virus entry and infection (Fig 7). This suggests that SIRPA is a host protein that could be targeted at the early stages of infection to inhibit infection of human pathogenic viruses.

## Materials and methods

### Ethics statement

All mice were housed according to the policies of the Animal Care Committee of the University of Illinois at Chicago; all studies were performed in accordance with the recommendations in the Guide for the Care and Use of Laboratory Animals of the National Institutes of Health. The experiments performed with mice in this study were approved by University of Illinois at Chicago ACC protocol #18–168.

### Mice

Strain A *Trim 2* KO mice were previously described [4]. B6.129P2 Sirpa<tm1Nog>/Rbrc (*Sirpa* KO) mice, which lack exons 7 and 8 in the *Sirpa* gene, were purchased from the Riken Institute (Japan) [18]. Genotyping was carried out according to the methods detailed in the Riken website https://mus.list.brc.riken.jp/ja/wp-content/pdf/01544_PCR.pdf. Both TRIM2 and SIRPA KO mice were maintained as heterozygotes. WT controls were derived from these crosses.

### Cell lines

Vero E6, U2OS, BV-2, NR-9456, A549 and 293T cells were grown in Dulbecco’s modified Eagle Medium (DMEM; Gibco) supplemented with 2 mM glutamine, 10% fetal bovine serum (FBS, Invitrogen) and penicillin (100 U/ml)-streptomycin (100 μg/ml) (Invitrogen) (DMEM complete). NR-9456 cells were cultured in the same media containing 0.1% sodium pyruvate, while 293T cells stably expressing human ACE2 (293T-ACE2) and mouse mCAT-1 (293T-mCAT) were cultured with puromycin (500 ng/ml) and geneticin (1 μg/ml), respectively.

### RNA interference

The following siRNAs were used for gene depletion: human SIRPA (Ambion #4392420), human ACE-2 (Qiagen #SI00131194), human TIM-1 (Dharmacon #J-019856-06-0002), human TRIM2 (Qiagen #SI04165602), mouse SIRPA (Dharmacon #D-042804-04-0002), siRNA control (Qiagen #1022076); siRNAs from Ambion (catalog no. 4392420) were used together to deplete expression of the VGCC (A1S, siRNA ID: s2297; A2D2, siRNA ID:214263; B3, siRNA ID: 105411). Briefly, cells were transfected at 60–70% confluence with the indicated siRNAs using the Lipofectamine RNAi Max (Invitrogen) forward transfection method for 48 hr. Knockdowns were verified by RT-qPCR with the primers described in Table A in S1 Text. Cells were infected for 1–2 hr at 37°C with infectious viruses or pseudoviruses and incubated for another 14–48 hr, as indicated in the figure legends, before measuring viral RNA/DNA levels by RT-qPCR or luciferase activity.

### Viruses

JUNV-C1 and VSV-eGFP were propagated in Vero E6 cells; LCMV (Armstrong strain) and Tacaribe virus (TRVL-11573; BEI Resources) were propagated in BHK-21 cells. Cell monolayers were infected at 70–80% confluence at a MOI of 0.01–0.03. Medium was removed at 24 hpi, the cells were washed with phosphate-buffered saline (1X PBS) and fed with DMEM containing 2% FBS. Virus particles were collected at 7–10 dpi and were purified by centrifugation through a 30% sucrose cushion, resuspended in 2% DMEM or 1X PBS and stored at -80°C until use. Moloney MLV was harvested from the supernatants of stably infected NIH 3T3 fibroblasts [53]. The supernatant was passed through a 0.45 μm filter and treated with 20U/ml DNase-1 (Roche) at 37°C for 30 min. HSV-1 (KOS strain; ATCC VR1493) was grown on Vero E6 cells. Briefly, cells were inoculated at a MOI of 0.01 for 2h and cultured for 2–3 days, until 100% of cells displayed cytopathic effect (CPE). At 100% CPE, the flask was frozen at -80°C, thawed at 37°C and the cell suspension was sonicated 3 times for 30 seconds to free virus particles. Zika virus (ZIKV) strain PF13 was obtained from Michael Diamond, Washington University. Viral stocks were propagated in C6/36 cells and culture supernatants were harvested at 6 dpi. Murine norovirus (mNoV) strain CW3 (obtained from Donna MacDuff, UIC) was grown in BV-2 cells by infecting 80–90% confluent cells at a MOI of 0.05 for 36–48 hr. Tissue culture flasks were frozen at -80° C, thawed at 37°C, culture suspensions were centrifuged at 3000 rpm for 20 min at 4°C, filtered (0.45 μm) and concentrated with Vivaflow 50 system filters (Sartorius).

### Virus titration

JUNV-C1 titers were determined by infectious center assays (ICA), TCRV was titrated by TCID_50_, and LCMV and VSV titers were determined by plaque assays in Vero E6 cells, as previously described [25]. MLV titers were determined by a focus-forming assay (FFA) on NIH 3T3 cells by using monoclonal anti-ENV (Ab538) antibody, as previously described [23]. HSV-1 titers were determined by plaque assay in Vero E6 cells as follows: 80–90% confluent monolayers were infected with serial dilutions of the virus for 2h at 37, the inoculum was then removed and replaced with 2% FBS, 5% methylcellulose media, and 3 dpi cells were fixed with methanol and stained with 0.1% crystal violet. ZIKV stock titers were determined by FFA using a monoclonal antibody (clone ZV67) obtained from Michael Diamond. mNoV titers were determined by plaque assay at 3 dpi, using 10 g/L methylcellulose and 10% FBS overlays.

### Pseudoviruses

HIV/EBOV and HIV/SARS-2 pseudoviruses were generated by transient co-transfection of the replication-defective HIV vector (pNL4-3.Luc.RE) and plasmids encoding the EBOV GP (pCAGGS, EBOV Zaire Mayinga strain Glycoprotein) or SARS-CoV-2 S (pCAGGS, SARS-CoV-2 Wuhan-Hu-1 strain Spike protein; BEI NR-52310) proteins into 293T cells, using a polyethylenimine-based transfection protocol [54]. Six hours after transfection, the cells were washed with PBS and 20 ml of fresh media were added to each 150 mm plate. Twenty-four hr post-transfection, the supernatants were collected and filtered through 0.45 μm pore filters (Nalgene). MLV-based pseudoviruses encoding the luciferase gene and bearing MACV GP and VSV G proteins were made as previously described [55].

### In vitro and ex vivo infections

U2OS and 293T-ACE2 cells were infected with replication-competent viruses at a MOI of 0.1 for 14–48 hpi as indicated in the figure legends, and viral RNA or DNA was isolated to assess infection levels by RT-qPCR or qPCR, respectively. MLV infections of 293-mCAT cells were done at a MOI of 0.1 and viral DNA was measured by qPCR at 48 hpi. mNoV infections of NR9456 cells were also carried out with a MOI of 0.1 and viral RNA was determined at 14 hpi. MLV- and HIV-based pseudovirus infections were performed for 48 hr. and luciferase activity was quantified using the Britelite plus Reporter Gene Assay System (Perkin Elmer). Bone marrow-derived mouse macrophages were infected with infectious viruses at a MOI of 1 and the cells were harvested at 24–48 hpi to assess infection levels by RT-qPCR.

### Generation of mouse primary cells

BMDMs were isolated from the hind limbs of 8-12-week-old SIRPA KO, TRIM2 KO and WT mice as described [56]. Macrophages were cultured in complete DMEM supplemented with 100 μg/ml of macrophage colony–stimulating factor (M-CSF; Gibco). Cells were harvested 7 days after plating and seeded in 24-well plates for virus infections and fluorescence-activated cell sorting (FACS) analysis, and in 6-well plates for immunoblotting. Ear fibroblasts from 8-12-week-old SIRPA KO and WT mice were cultured in DMEM complete and infected as previously described [23].

### Western blots

Protein extracts (50 μg) and JUNV-C1 virus particles (5E5 ICs) were resolved on 4–15% gradient SDS-polyacrylamide gels and transferred to polyvinylidene difluoride (PVDF) membranes (Millipore). FLAG-tagged SIRPA constructs were detected using a mouse anti-FLAG (M2) (SIGMA #F1804), the cytoplasmic domain of mouse SIRPA was detected using a rabbit polyclonal antibody (CST #13379S) and the ectodomain of mouse SIRPA was detected with a rat monoclonal antibody (clone p84; Biolegend #14401). Detection of JUNV nucleoprotein (NP) was done using the mouse monoclonal antibody NR-2582 (BEI Resources; clone NA05-AG12) and CD47 was detected with the B6H12 monoclonal antibody (BioXCell; BE0019-1). Human ACE2 was detected with a mouse monoclonal antibody (#66699-1-Ig; Proteintech). GAPDH and β-actin served as internal controls and were detected using a rabbit polyclonal (CST #2118L) and a mouse monoclonal (Proteintech # 66009-1-Ig), respectively.

### Phagocytosis assay

Thymocytes from a C57BL/6N mouse were treated with 0.1 μM dexamethasone (Sigma) for 14 hr at 37°C to induce apoptosis and then stained with pHrodo Red, succinimidyl ester (ThermoScientific) for 1 hr. Fully differentiated BMDMs from WT and SIRPA KO mice (2 mice each) were incubated in duplicate with 1mM MnCl_2_ (SIGMA) and fed apoptotic thymocytes at a ratio of 1:5 for 2 hr at 37°C. The cells were detached with 1mM EDTA, stained with APC-conjugated anti-CD11b (Biolegend) for 1hr at 4°C and analyzed by FACS. The percentage of double-positive cells was determined, and the percent of engulfment was normalized to WT cells for each experiment. Presented is the average of 2 independent experiments.

### In vivo infections

Eight-to-twelve-week-old mice were inoculated with 1x10^6^ ICs JUNV-C1 by intraperitoneal injection. Mice were sacrificed at 7 and 14 dpi, and the spleens were harvested for viral RNA isolation and virus titration. Neonatal mice (1–3 days old) were infected with 2x10^3^ TCID_50_ of TCRV by intraperitoneal inoculation. One-week post-infection, TCRV RNA levels and titers in spleen were analyzed. One-to-2-day-old mice were infected by intraperitoneal injection of 2x10^3^ ICs of Moloney MLV and spleens were harvested at 16 dpi, as previously described [22]. Six-week-old mice were orally infected with 1x10^6^ PFU mNoV and at 3 dpi spleens were harvested to analyze viral RNA.

### Nucleic acid isolation and RT-qPCR

DNA was isolated with the DNeasy Blood & Tissue Kit (Qiagen). Total RNA was isolated using the RNeasy kit (Qiagen) for cell cultures or TRIzol Reagent (Invitrogen) for tissues. RNA was used as a template for cDNA synthesis (SuperScript III First-Strand Synthesis System; Invitrogen) in a reaction mixture primed with random hexamers (50 ng/μl). Viral and cellular RNAs were detected by RT-qPCR using a QuantStudio 5 Real-Time PCR System (Applied Biosystems) with specific primer pairs (Table A in S1 Text). RNA was normalized to glyceraldehyde-3-phosphate dehydrogenase (GAPDH). RT-qPCR reactions were done using Power SYBR Green Master Mix (Applied Biosystems). The amplification conditions were as follow: 50°C for 2 min, 95°C for 10 min, and 40 cycles of 95°C for 15 s and 60°C for 1 min. The efficiency of amplification was determined for each primer set by a standard curve with 10-fold serial dilutions of DNA of known concentration. The slope values of the standard curves for the primer pair amplicons ranged from 3.5 to 3.2. For each primer pair, a non-template control was included, and each sample was run in triplicate.

### FACS analysis

Adherent cells were dispersed with 1X PBS/1mM EDTA. The following antibodies were used for staining: FITC anti-human SIRPA (CD172a; eBioscience), mouse monoclonal anti-human CD47 (BioXCell), rat monoclonal anti-mouse SIRPA (CD172a; clone p84; Biolegend). Alexa Fluor -488 and -647 conjugated were used as secondary antibodies (Thermo Scientific). The cells were stained and washed with FACS buffer (1% FBS 0.01% sodium azide in 1X PBS), and were analyzed in a BD LSRFortessa cell analyzer (BD Biosciences) using FlowJo v10 software (Tree Star, Inc.).

### Binding assay using FITC-labeled viruses

TCRV was concentrated by centrifugation on 30% sucrose cushions, resuspended in sterile 1X PBS, titrated, and labeled with FITC using the Fluorotag FITC conjugation kit (Sigma). A MOI of 10 was used for experiments. SARS-2-S pseudoviruses were treated with DNaseI (20U/ml for 45min at 37°C) (Roche), concentrated by sucrose cushion, labeled with FITC and used for the infections at a MOI of 20. Cells were incubated with FITC-labeled viruses for 1 hr on ice and virus binding was assayed by flow cytometry.

### Virus internalization assay

Primary cells or cell lines were incubated on ice with JUNV-C1, TCRV, LCMV, VSV and mNoV (MOI = 5) for 1 hr, shifted to 37°C for an additional 45 min, treated with 1 mg/ml of Proteinase K (Gibco) for 45 min on ice to strip virus from the cell surface, followed by treatment with 2mM of the inhibitor phenylmethylsulfonyl fluoride (SIGMA) to inactivate Proteinase K. Internalized viral RNA was isolated and used for RT-qPCR.

### CD47 blocking assay

Surface CD47 was blocked using a mouse monoclonal anti-human CD47 (clone B6.H12, BioXCell #BE0019-1). Mouse IgG1 (BioXCell #BE0083) and Pooled Non-Human Primate Convalescent Serum to SARS-CoV-2 (NR-52401, BEI Resources) were used as negative and positive controls, respectively. In brief, SARS-2-S and VSV-G pseudoviruses, produced in 293T cells, and JUNV-C1 and LCMV isolated from infected U2OS cells, were incubated with 10 μg/ml of CD47 antibody or mouse IgG1 isotype control or anti-SARS-CoV-2 serum for 1 hr on ice. The antibody-treated viruses and pseudoviruses were used to infect U2OS and 293T-ACE2 cells, respectively. Viral RNA or luciferase activity was measured at 24 and 48 hpi, respectively.

### Chemicals and biologicals

Camostat mesylate, E-64, and nanchangmycin were purchased from SIGMA and reconstituted following the manufacturer’s instructions. Adherent cells were treated with 25 μM camostat, 100 μM E-64, 100 nM nanchangmycin or DMSO for 2 hr at 37°C. After treatment, the medium was removed, and the cells were infected with indicated pseudoviruses. Luciferase activity was measured at 48 hpi. MnCl_2_ tetrahydrate was purchased from SIGMA and was diluted in ddH_2_O. Cell lines and primary cells were treated with 1mM MnCl_2_ for 2 hr, and subjected to viral infections, VIAs and western blot. Recombinant human IL-4 (InvivoGen) was reconstituted in ddH_2_O and used at different concentrations to stimulate U2OS cells for 48 hr prior to viral infections, VIAs and western blot.

### Generation of SIRPA expression constructs

Human full-length SIRPA construct was previously described [4]. The ΔCyto construct was generated by PCR using the human SIRPA plasmid as template and the primers SIRPA-Clo-For and ΔCyto-Rev (Table B in S1 Text); a FLAG-tag was included in the ΔCyto-Rev primer. The 1-4A SIRPA mutant was generated by PCR-based site-specific mutagenesis using the SIRPA full-length construct as template and the Q5 Site-Directed Mutagenesis Kit (New England Biolabs). The primers used to generate the 1–4 A mutant are detailed in Table B in S1 Text. All constructs were validated by Sanger sequencing.

### DNA transfection

DNA expression plasmids were transfected into 80–90% confluent 293T cells seeded in six-well plates for 24 hr using Lipofectamine 3000 reagent (Thermo Scientific), according to the manufacturers’ instructions. Cells were lysed with 500 μl of 1X cell lysis buffer (CLB; Cell Signaling Technologies [CST]) supplemented with 2% of Halt Protease and Phosphatase Inhibitor Cocktail (Thermo Scientific).

### Statistical analysis

Each experiment was done with 3 technical replicates per experiment. Data shown is the average of at least 3 independent experiments, or as indicated in the figure legends. Statistical analysis was performed using the GraphPad 8.1/PRISM software. Tests used to determine significance are indicated in the figure legends.

## Supporting information

S1 Fig SIRPA knockdown validation in cell lines A) used for virus (Fig 1A) and B) pseudovirus infection (Fig 1B). Shown is the average of 2 experiments.
(TIF)

S2 Fig A) Detection of SIRPA in 293T-ACE2, Calu-3 and U2OS cells using a rabbit polyclonal anti-SIRPA; anti-GAPDH served as a control. B) Knockdown validation of SIRPA and ACE2 RNA levels in 293T-ACE2 and Calu-3 cells (Fig 2). Shown is the average of 2 experiments.
(TIF)

S3 Fig Western blot analysis of FLAG-tagged SIRPA WT and mutant constructs expression in transiently transfected cells. A) SIRPA WT and ΔCyto in 293-ACE2 cells; ACE2 was detected using a mouse monoclonal. B) SIRPA WT, 1-4A and ΔCyto expression in U2OS cells. Constructs were detected using a mouse anti-FLAG antibody. Anti-GAPDH served as a control.
(TIF)

S4 Fig Surface expression of SIRPA and CD47 in 293T and U2OS cells; cells were stained with a FITC-conjugated anti-human SIRPA (CD172a) antibody and B6H12 followed by an Alexa Fluor 647-conjugated secondary antibody. (TIF)

S5 Fig SIRPA expression upon MnCl_2_ treatment in U2OS cells. A) RNA levels. Shown is the average of 6 experiments. One-way ANOVA was used to determine significance. **, *P* ≤ 0.003; ****, *P* ≤ 0.0001 B) Detection of endogenous SIRPA by western blot.
(TIF)

S6 Fig MnCl2 treatment enhances phagocytosis by WT BMDMs to the same levels as SIRPA KO BMDMs. A) Representative FACS plots. B) Quantification of 3 independent assays. Significance was determined by one-way ANOVA. *, *P* ≤ 0.01; **, *P* ≤ 0.002.
(TIF)

S7 Fig A) Binding assay of TCRV-FITC (U2OS cells) and SARS-2-S-FITC (293T-ACE2 cells). B) siRNA-mediated knockdown validation in cells used for VIAs. Shown is the average 3 experiments. Unpaired t test was used to determine significance. **, *P*≤0.009; *** *P*≤ 0.0002; ****, *P*≤0.0001.
(TIF)

S8 Fig A) Surface expression of SIRPA in U2OS cells upon IL-4 treatment for 48 hr. B) Median fluorescence intensity (MFI) of SIRPA from 3 independent experiments. Significance was determined by one-way ANOVA. **, *P* ≤ 0.004; ***, *P* ≤ 0.0008.
(TIF)

S1 Text Tables. A. Primer pairs used for reverse-transcribed RT-qPCR. B. Primer pairs used for PCR-mediated mutagenesis and molecular cloning of SIRPA constructs. The primer positions are based on the SIRPA reference sequence NM_001040022.1. In bold is depicted the sequence corresponding to SIRPA, in italics the sequence of the FLAG tag, with underlines, the coding sequences mutated from tyrosine to alanine and in red, the unmutated codon.
(DOCX)
