# Supplementary figures and images for: Signal-regulatory protein alpha is an anti-viral entry factor targeting viruses using endocytic pathways
Source: PLoS Pathog. 2021 Jun 7;17(6):e1009662. doi: 10.1371/journal.ppat.1009662 (PMC8211255; doi:10.1371/journal.ppat.1009662)

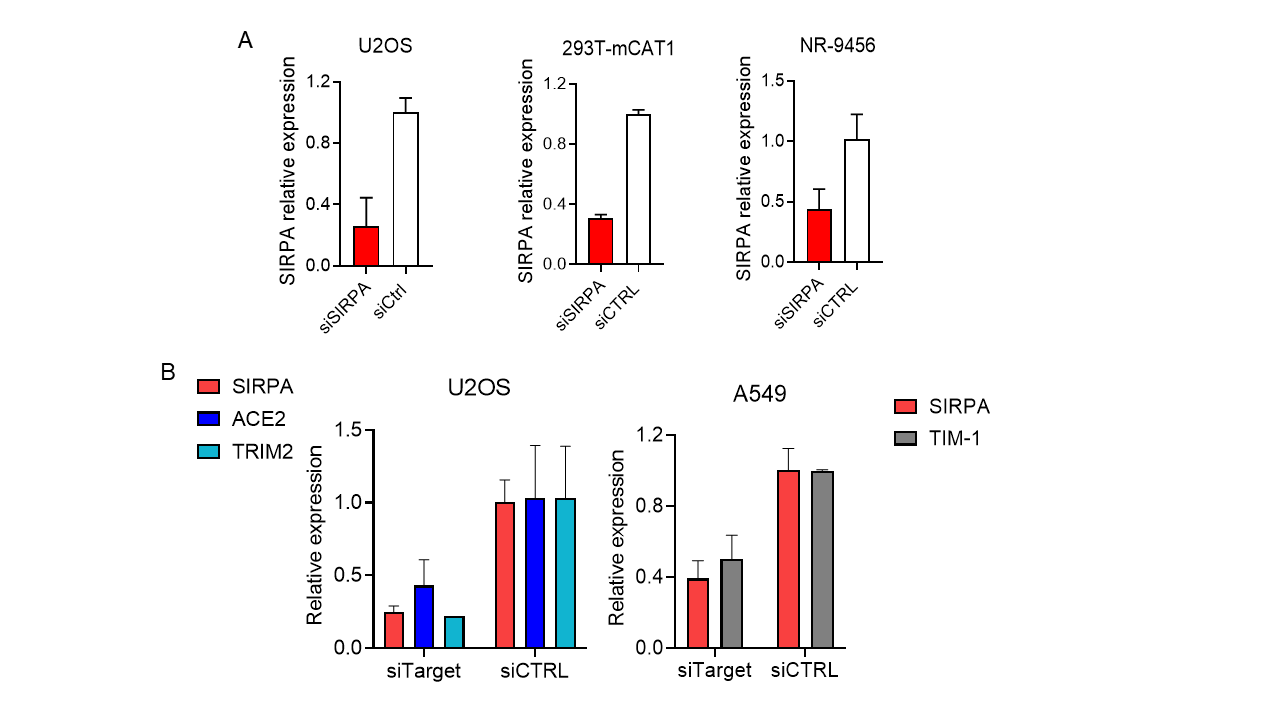

Supplement: S1 Fig — SIRPA knockdown validation in cell lines A) used for virus (Fig 1A) and B) pseudovirus infection (Fig 1B). Shown is the average of 2 experiments. (TIF) [file ppat.1009662.s001.tif]

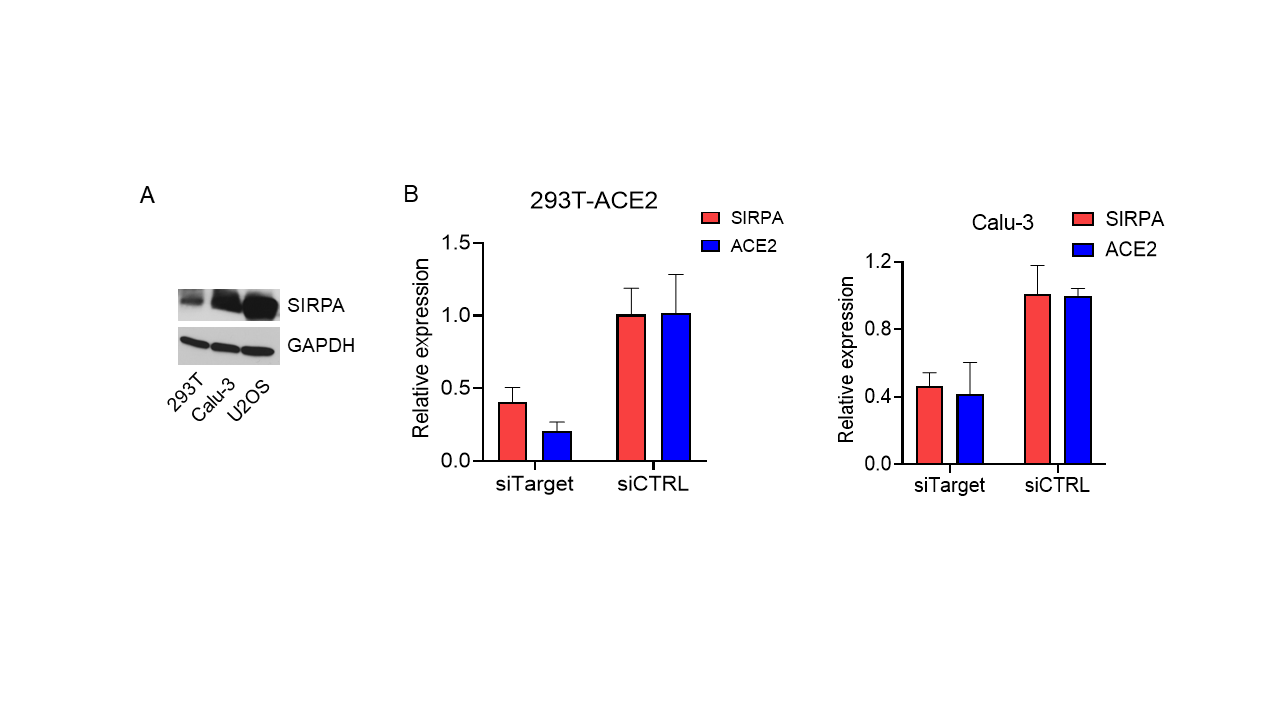

Supplement: S2 Fig — A) Detection of SIRPA in 293T-ACE2, Calu-3 and U2OS cells using a rabbit polyclonal anti-SIRPA; anti-GAPDH served as a control. B) Knockdown validation of SIRPA and ACE2 RNA levels in 293T-ACE2 and Calu-3 cells (Fig 2). Shown is the average of 2 experiments. (TIF) [file ppat.1009662.s002.tif]

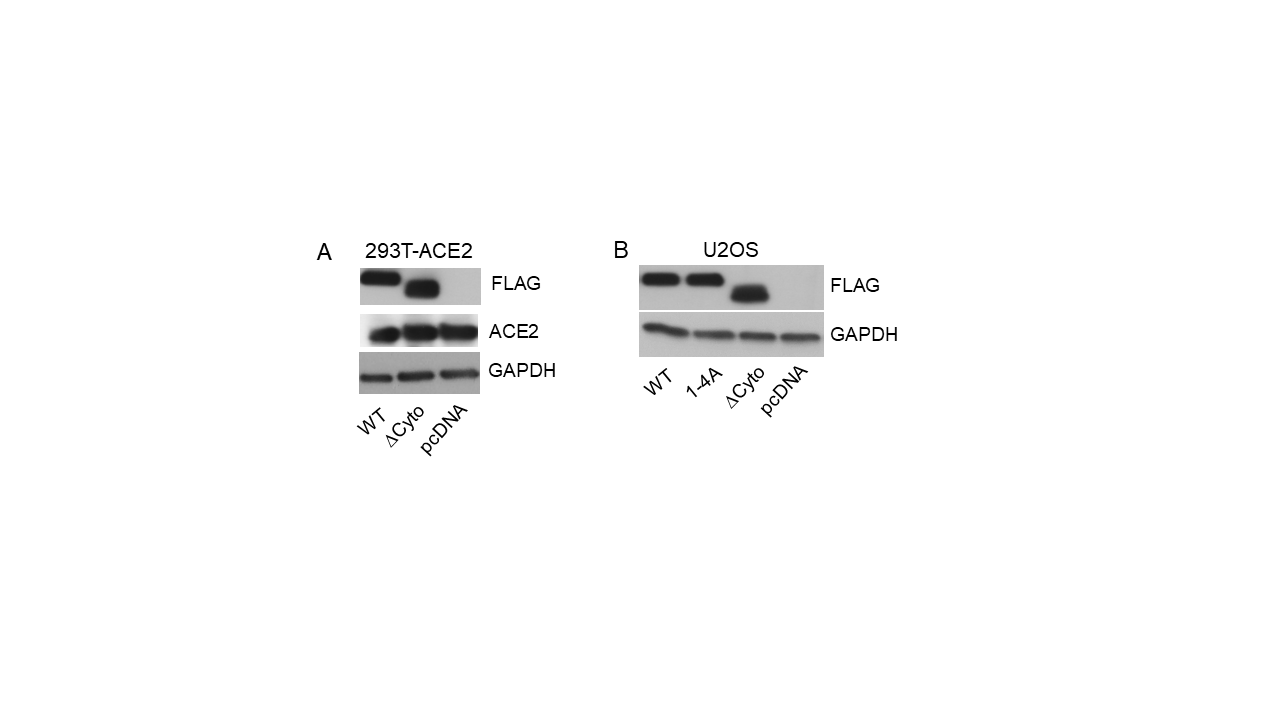

Supplement: S3 Fig — A) SIRPA WT and ΔCyto in 293-ACE2 cells; ACE2 was detected using a mouse monoclonal. B) SIRPA WT, 1-4A and ΔCyto expression in U2OS cells. Constructs were detected using a mouse anti-FLAG antibody. Anti-GAPDH served as a control. (TIF) [file ppat.1009662.s003.tif]

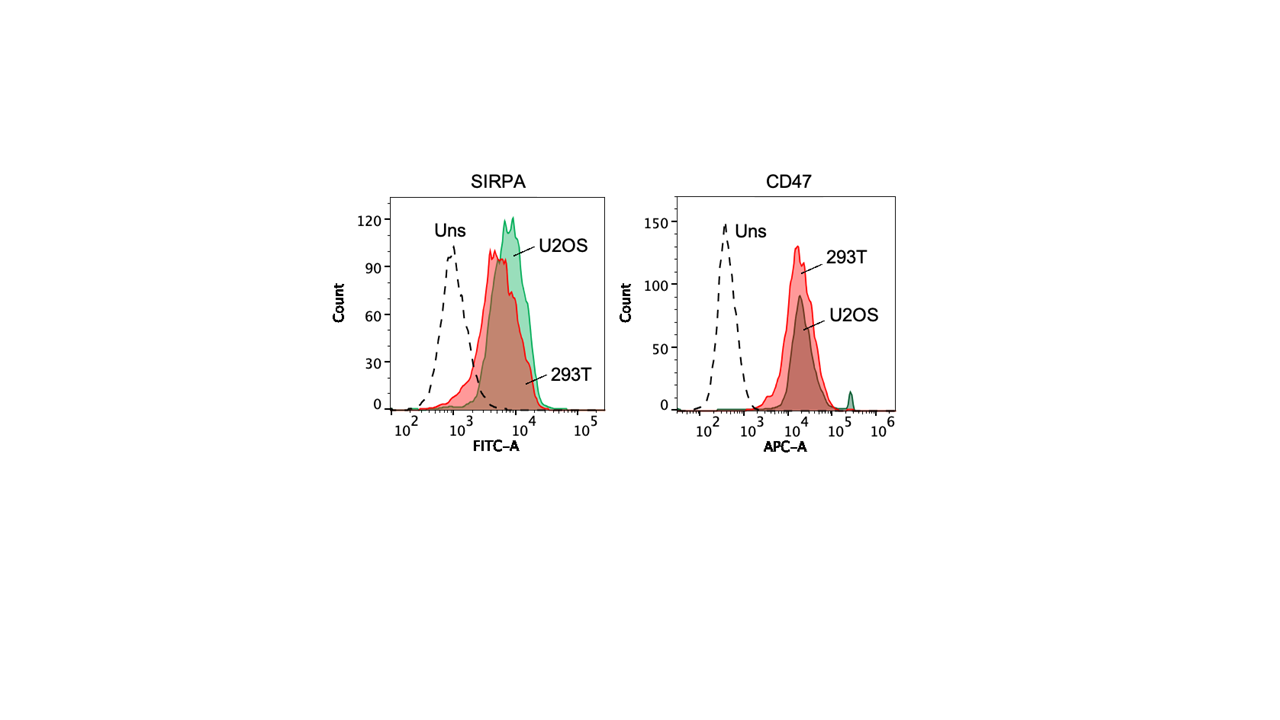

Supplement: S4 Fig — (TIF) [file ppat.1009662.s004.tif]

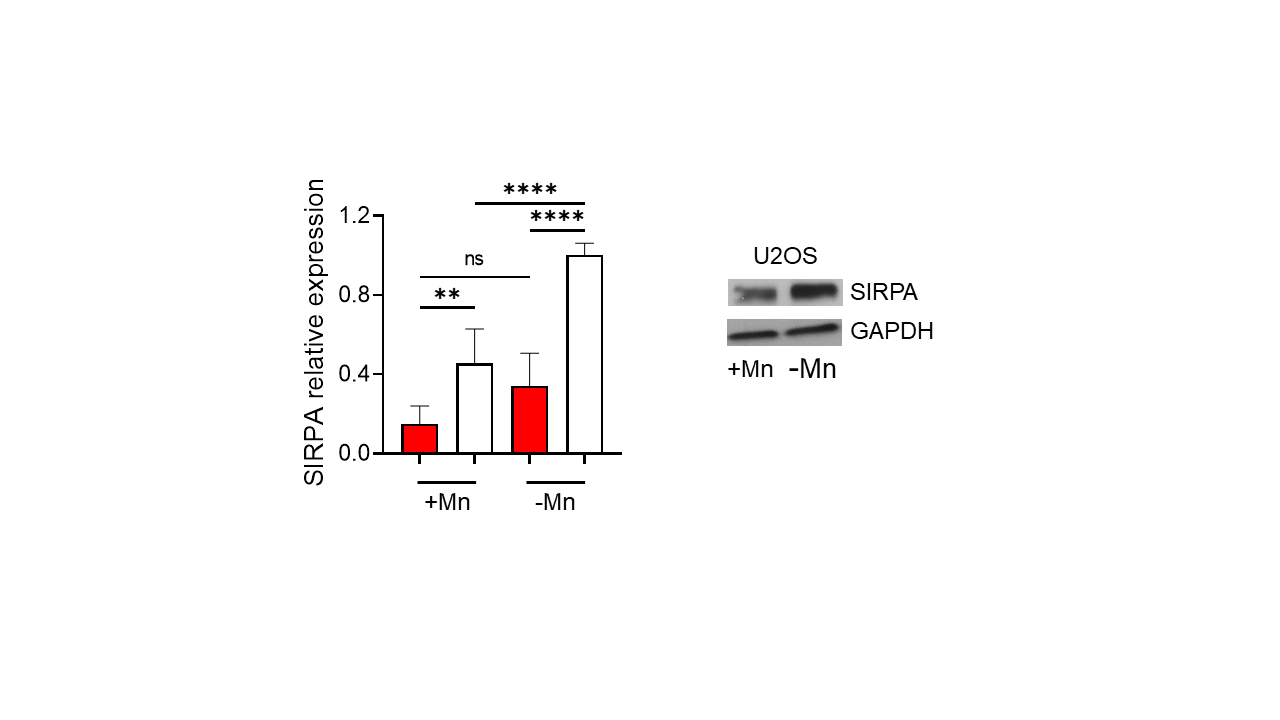

Supplement: S5 Fig — A) RNA levels. Shown is the average of 6 experiments. One-way ANOVA was used to determine significance. **, P ≤ 0.003; ****, P ≤ 0.0001 B) Detection of endogenous SIRPA by western blot. (TIF) [file ppat.1009662.s005.tif]

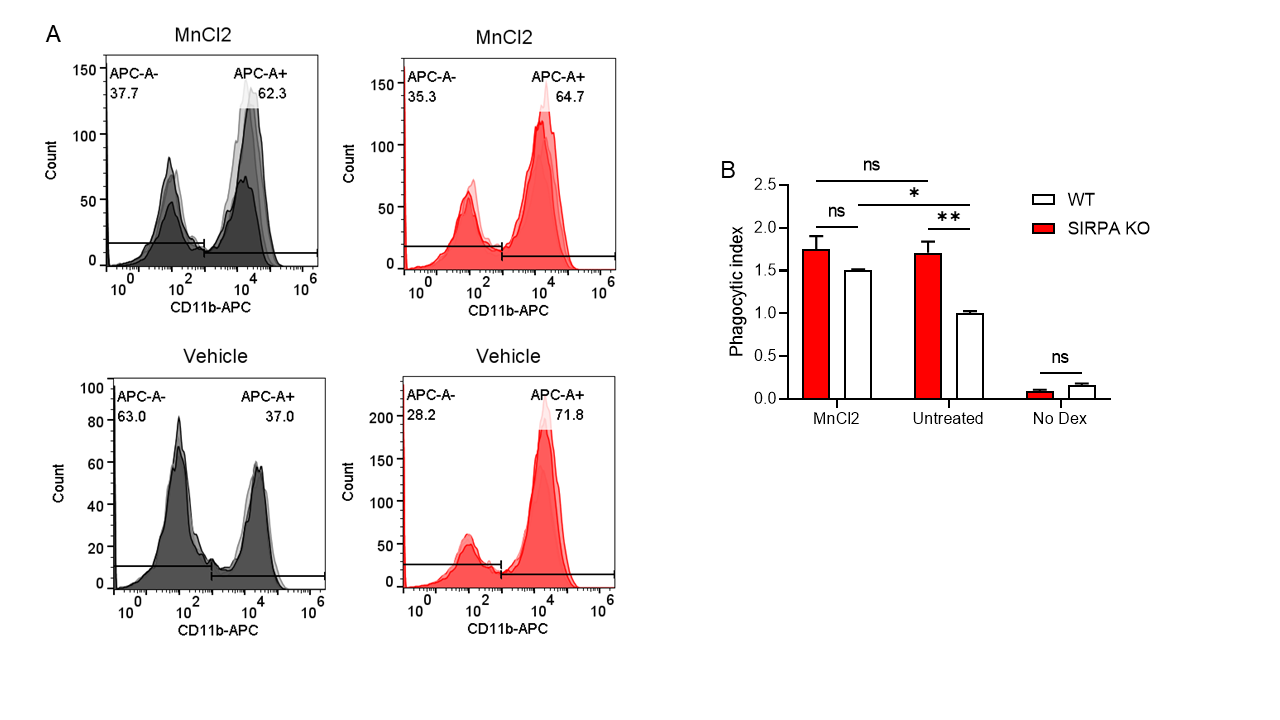

Supplement: S6 Fig — A) Representative FACS plots. B) Quantification of 3 independent assays. Significance was determined by one-way ANOVA. *, P ≤ 0.01; **, P ≤ 0.002. (TIF) [file ppat.1009662.s006.tif]

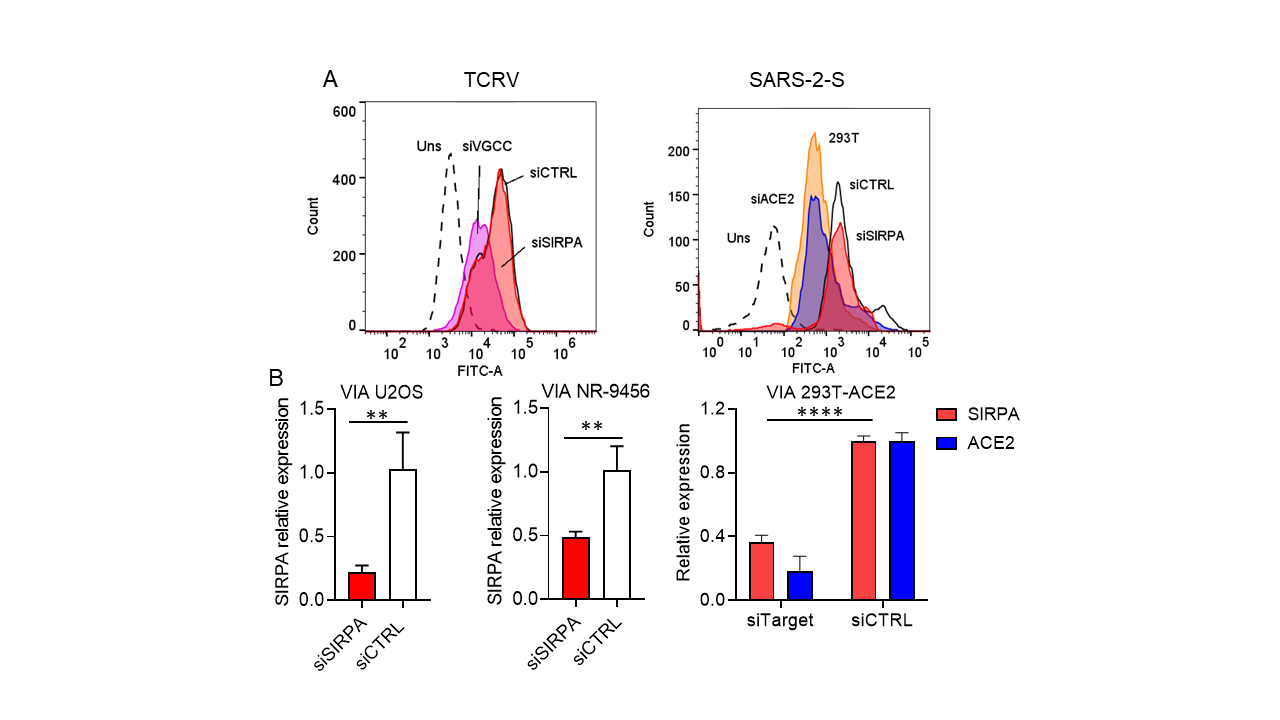

Supplement: S7 Fig — A) Binding assay of TCRV-FITC (U2OS cells) and SARS-2-S-FITC (293T-ACE2 cells). B) siRNA-mediated knockdown validation in cells used for VIAs. Shown is the average 3 experiments. Unpaired t test was used to determine significance. **, P≤0.009; *** P≤ 0.0002; ****, P≤0.0001. (TIF) [file ppat.1009662.s007.tif]

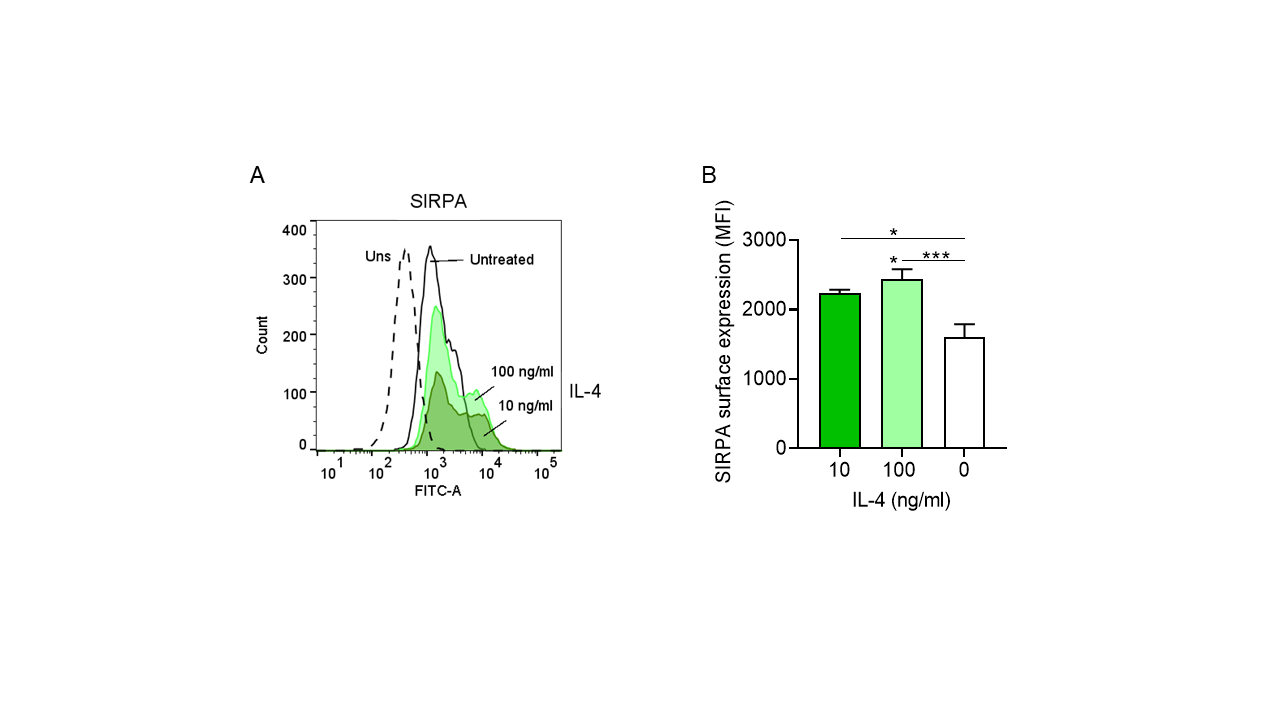

Supplement: S8 Fig — A) Surface expression of SIRPA in U2OS cells upon IL-4 treatment for 48 hr. B) Median fluorescence intensity (MFI) of SIRPA from 3 independent experiments. Significance was determined by one-way ANOVA. **, P ≤ 0.004; ***, P ≤ 0.0008. (TIF) [file ppat.1009662.s008.tif]
