# Supplementary material for: Signal-regulatory protein alpha is an anti-viral entry factor targeting viruses using endocytic pathways
Source: PLoS Pathog. 2021 Jun 7;17(6):e1009662. doi: 10.1371/journal.ppat.1009662 (PMC8211255; doi:10.1371/journal.ppat.1009662)
Supplement: S1 Text — A. Primer pairs used for reverse-transcribed RT-qPCR. B. Primer pairs used for PCR-mediated mutagenesis and molecular cloning of SIRPA constructs. The primer positions are based on the SIRPA reference sequence NM_001040022.1. In bold is depicted the sequence corresponding to SIRPA, in italics the sequence of the FLAG tag, with underlines, the coding sequences mutated from tyrosine to alanine and in red, the unmutated codon. (DOCX) [file ppat.1009662.s009.docx]

Table A. Primer pairs used for reverse-transcribed RT-qPCR.

| JUNV-SF | GGG GCA GTT CAT TAG CTT CAT GC |
| --- | --- |
| JUNV-SR | CAA AGG TAG GTC ATG TGG ATT GTT GG |
| LCMV-SF | AGA ATC CAG GTG GTT ATT GCC |
| LCMV-SR | GTT GTA GTC AAT TAG TCG CAG C |
| VSV-NP-F | TGA ATG TGC CTC GTT CAG ATA |
| VSV-NP-R | CCA AAG TCG ATC AAA TAA GGC |
| GAPDH-F | CCC CTT CAT TGA CCT CAA CTA CA |
| GAPDH-R | CGC TCC TGG AGG ATG GTG AT |
| TCRV-GPF | TCG GTC ACA GAT GGG ACC AGG |
| TCRV-GPR | CAG GGT TCT TCA CGT CCT CTG |
| A2D2-F | TGC CAA TCA GCA AAC TGA AG |
| A2D2-R | CCC GTG TCC AGA ATC AAG TT |
| TIM-1-F | TGT GCT GGA ATA GAG GCT CAT |
| TIM-1-R | CAA TAT ACG CCA CTG TCA GAC |
| ZIKV-prE-F | CAG CAA TAG AGA CTT CGT GGA |
| ZIKV-prE-R | CAG CAA TAG AGA CTT CGT GGA |
| hSIRPA-F | TTC CAG TGC CTT CCA GCC CT |
| hSIRPA-R | GGT GAT GTT ACC GAT GCG GAT G |
| mSIRPA-F | CAT CCA GCC AGC CAA TCC TGT |
| mSIRPA-R | TCC AGT TCG CCC TCT GGT TCT |
| TRIM2-F2 | TGT CTG CAC ACT TTC TGC GAG AG |
| TRIM2-R2 | GTT TGG GCA CGA AAG AGG CTT TC |
| hACE2-F | GGA CTC TGC CAT TTA CTT ACA |
| hACE2-R | CAA CTA TCT CTC GCT TCA TCT |
| ffLuc-F | CAC ATA TCG AGG TGA ACA TCA |
| ffLuc-R | AGG TAG ATG AGA TGT GAC GAA |
| GAP DNA-F | CCC CTT CAT TGA CCT CAA CTA CA |
| GAP DNA-R | CGC TCC TGG AGG ATG GTG AT |
| suMLV-F | CCT ACT ACG AAG GGG TGG |
| suMLV-R | CAC ATG GTA CCT GTA GGG GC |
| HSV1-TK-F | GAG TTT CAC GCC ACC AAG AT |
| HSV1-TK-R | CTA TGA TGA CAC AAA CCC CG |
| mNoV-F | CAC GCC ACC GAT CTG TTC TG |
| mNoV-R | GCG CTG CGC CAT CAC TC |

Table B. Primer pairs used for molecular cloning of SIRPA ΔCyto construct and PCR-mediated mutagenesis. The primer positions are based on the SIRPA reference sequence NM_001040022.1. In bold is depicted the sequence corresponding to SIRPA, in italics the sequence of the FLAG tag, with underlines, the coding sequences mutated from tyrosine to alanine and in red, the unmutated codon.

| Primer | Position in cDNA |  |
| --- | --- | --- |
| SIRPA-Clo-For | 1-18 | TAA TGG GGA TCC GCA **ATG GAG CCC GCC GGC CCG** |
| SIRPA-ΔCyto-Rev | 1241-1225 | TTG TTG TCT AGA *CTT GTC ATC GTC TTT GTA GTC* **CTG GGC TTT CTT CTG TCT** |
| hSIRPA-1YA-For | 1274-1298 | **TGA TAT CAC AGC TGC AGA CCT GAA C**  TA T |
| hSIRPA-1YA-Rev | 1273-1254 | **TTT GTG TCC TGT GTT ATT TC** |
| hSIRPA-2YA-For | 1346-1373 | **CCA CAC GGA GGC TGC CAG CAT TCA GAC C**  TA T |
| hSIRPA-2YA-Rev | 1345-1328 | **TTG TTG GGC TCC GCA GCC** |
| hSIRPA-3YA-For | 1397-1431 | **CAC CCT CAC CGC TGC TGA CCT GGA CAT GGT CCA C**  TA T |
| hSIRPA-3YA-Rev | 1395-1379 | **TCC TCC GAC GCG GGC TGC** |
| hSIRPA-4YA-For | 1475-1500 | **CTT CTC AGA GGC CGC CAG CGT CCA GG**  TA C |
| hSIRPA-4YA-Rev | 1474-1457 | **GAC GGC TCA GGC TTG GGG** |
